# An order restricted multi‐arm multi‐stage clinical trial design

**DOI:** 10.1002/sim.9314

**Published:** 2022-01-19

**Authors:** Alessandra Serra, Pavel Mozgunov, Thomas Jaki

**Affiliations:** ^1^ MRC Biostatistics Unit University of Cambridge Cambridge UK; ^2^ Department of Mathematics and Statistics Lancaster University Lancaster UK

**Keywords:** adaptive designs, infectious diseases, multi‐arm multi‐stage, order restriction

## Abstract

One family of designs that can noticeably improve efficiency in later stages of drug development are multi‐arm multi‐stage (MAMS) designs. They allow several arms to be studied concurrently and gain efficiency by dropping poorly performing treatment arms during the trial as well as by allowing to stop early for benefit. Conventional MAMS designs were developed for the setting, in which treatment arms are independent and hence can be inefficient when an order in the effects of the arms can be assumed (eg, when considering different treatment durations or different doses). In this work, we extend the MAMS framework to incorporate the order of treatment effects when no parametric dose‐response or duration‐response model is assumed. The design can identify all promising treatments with high probability. We show that the design provides strong control of the family‐wise error rate and illustrate the design in a study of symptomatic asthma. Via simulations we show that the inclusion of the ordering information leads to better decision‐making compared to a fixed sample and a MAMS design. Specifically, in the considered settings, reductions in sample size of around 15% were achieved in comparison to a conventional MAMS design.

## 1 INTRODUCTION

Drug development is costly and time consuming.^1^ One family of clinical trial designs that can improve the development process are multi‐arm multi‐stage designs (MAMS).^2^, ^3^, ^4^ In a MAMS trial, insufficiently promising treatments can be dropped or the trial can be stopped due to overwhelming benefit at a series of interim analyses.

To date these designs have focused on the setting of independent treatment arms and have been argued to be a highly efficient approach to clinical trials.^5^, ^6^, ^7^ They could, however, be suboptimal if an “order” (ie, a monotonic relationship) among the treatment effects can be assumed. Such an order can occur naturally, for example, when multiple doses or administration schedules of the same treatment are tested or when nested combinations of treatments are investigated. Another area where an order can often be assumed is when considering different treatment durations. In infectious diseases such as Tuberculosis (TB) and Hepatitis B (HBV), the treatment duration with current standard regimes is lengthy^8^ which results in a large burden on the patients, potentially high costs, increased risk of non‐compliance and side effects.^9^ In TB and HBV, for example, treatment periods of 6 and 12 months are typical.^10^, ^11^ Novel treatments or combinations of treatments in these areas offer the opportunity for both higher efficacy and shorter treatment periods.^12^ In the setting of multiple treatment durations Quartagno et al^13^ have proposed to model the duration‐response curve. While this is an efficient way to understand the duration‐effect relationship, it is less clear how to definitively conclude whether a duration is “better” than the current standard.

In this work, we extend the MAMS framework and propose a design that incorporates the order of treatment effects in the decision‐making when no parametric dose‐response or duration‐response model is assumed. The objective of the design is to identify all promising arms (eg, treatment durations, doses, or combination of treatments), including the one associated with the smallest relevant treatment effect.

The rest of the manuscript continues as follows. A case study is introduced in Section 2 before a detailed description of the 3‐arm and 2‐stage design is provided in Section 3. Section 4 then generalizes the proposed design to an arbitrary number of arms and stages and provides some theoretical results. Section 5 revisits the case study before the design is evaluated via simulations in Section 6. In Section 7, the effect of various critical bounds on the operating characteristics of the proposed design is explored. We conclude with a discussion.

## 2 CASE STUDY SETTING

The *Tiotropium add‐on therapy in adolescents with moderate asthma: A 1‐year randomized controlled trial* (NCT01257230)^14^ is a Phase III study that assessed the efficacy and safety of once‐daily tiotropium via Respimat added to inhaled corticosteroid (ICS) with or without a leukotriene receptor antagonist in adolescent patients with moderate symptomatic asthma. Patients were randomized with equal probability to receive 5μg (2 puffs of 2.5μg) or 2.5μg (2 puffs of 1.25μg) of once‐daily tiotropium or placebo (2 puffs). The primary outcome was change from baseline in peak ${\text{FEV}}_{1}$ within 3 h after dosing (peak FEV${}_{1[0-3h]}$) measured after 24 weeks of treatment. The null hypotheses were tested in a stepwise manner to control the type I error starting from the highest dose suggesting that a monotonic dose‐response relationship can be assumed.

## 3 A 3‐ARM 2‐STAGE ORDER RESTRICTED DESIGN

In this section, we develop an order restricted design (ORD) for the setting of the case study. We denote the highest dose (5μg) by $T_{1}$ and the lower dose (2.5μg) by $T_{2}$. The generalization to an arbitrary number of arms and stages is given in Section 4.

Assume that a patient's response follows a normal distribution with known common variance, $\sigma^{2}$. An alternative approach is outlined in Section 8 for the case of unknown variance. Let $X_{i}^{\left(k\right)}∼N(\mu^{\left(k\right)},\sigma^{2}),\;k\in \{0,1,2\},i=1:n_{j}^{\left(k\right)}$ be the observation of the *i*th patient on treatment *k* (the control arm is denoted by 0) and $n_{j}^{\left(k\right)}$ be the number of patients on arm *k* up to stage *j*. Let $\theta^{\left(k\right)}=\mu^{\left(k\right)}-\mu^{\left(0\right)}$ be the true treatment effect of active arm k∈{1,2} compared to the control. We denote the vector of treatment effects by $\theta =(\theta^{\left(1\right)},\theta^{\left(2\right)})$. Consider the following order relationship: $\theta^{\left(1\right)}\geq \theta^{\left(2\right)}$, implying that the treatment effect of the second treatment is at most as large as the treatment effect for the first treatment. Let $r_{j}^{\left(k\right)}$ be the ratio between the number of subjects allocated to treatment k∈{0,1,2} and control at each stage *j* with $r_{j}^{\left(0\right)}=1$. Let $Z_{j}^{\left(k\right)}=\frac{{\hat{\mu }}_{j}^{\left(k\right)}-{\hat{\mu }}_{j}^{\left(0\right)}}{\sigma }\sqrt{\frac{r_{j}^{\left(0\right)}n_{j}^{\left(k\right)}}{r_{j}^{\left(k\right)}+r_{j}^{\left(0\right)}}}$ be the test statistic^3^ at stage *j* for comparing arm k∈{1,2} to control, where ${\hat{\mu }}_{j}^{\left(k\right)}={\left(n_{j}^{\left(k\right)}\right)}^{-1}\sum_{i=1}^{n_{j}^{\left(k\right)}}X_{i}^{\left(k\right)}$ and $n_{j}^{\left(k\right)}=r_{j}^{\left(k\right)}n$, with k∈{0,1,2} and *n* is the sample size in the control group at the first stage. The vector of test statistics follows a multivariate normal distribution $Z∼N_{4}(\epsilon ,\sum )$ with $Z=(Z_{1}^{\left(1\right)},Z_{1}^{\left(2\right)},Z_{2}^{\left(1\right)},Z_{2}^{\left(2\right)})$, $\epsilon =(\frac{\theta^{\left(1\right)}}{\sigma }\sqrt{\frac{r_{1}^{\left(0\right)}n_{1}^{\left(1\right)}}{r_{1}^{\left(1\right)}+r_{1}^{\left(0\right)}}},\frac{\theta^{\left(2\right)}}{\sigma }\sqrt{\frac{r_{1}^{\left(0\right)}n_{1}^{\left(2\right)}}{r_{1}^{\left(2\right)}+r_{1}^{\left(0\right)}}},\frac{\theta^{\left(1\right)}}{\sigma }\sqrt{\frac{r_{2}^{\left(0\right)}n_{2}^{\left(1\right)}}{r_{2}^{\left(1\right)}+r_{2}^{\left(0\right)}}},\frac{\theta^{\left(2\right)}}{\sigma }\sqrt{\frac{r_{2}^{\left(0\right)}n_{2}^{\left(2\right)}}{r_{2}^{\left(2\right)}+r_{2}^{\left(0\right)}}})$ and the covariances between *Z*‐statistics are $\text{Cov}(Z_{j}^{\left(k\right)},Z_{j}^{\left(k\right)})=1,$ with k,j∈{1,2}, $\text{Cov}(Z_{j}^{\left(k\right)},Z_{j}^{\left(k^{\prime }\right)})=\sqrt{\frac{r_{j}^{\left(k\right)}}{r_{j}^{\left(k\right)}+r_{j}^{\left(0\right)}}\frac{r_{j}^{\left(k^{\prime }\right)}}{r_{j}^{\left(k^{\prime }\right)}+r_{j}^{\left(0\right)}}},k\neq k^{\prime },$ with $k,k^{\prime },j\in \{1,2\}$, $\text{Cov}(Z_{1}^{\left(k\right)},Z_{2}^{\left(k\right)})=\sqrt{\frac{r_{1}^{\left(k\right)}r_{1}^{\left(0\right)}}{r_{1}^{\left(k\right)}+r_{1}^{\left(0\right)}}\frac{r_{2}^{\left(k\right)}+r_{2}^{\left(0\right)}}{r_{2}^{\left(k\right)}r_{2}^{\left(0\right)}}}$, with k∈{1,2}, $\text{Cov}(Z_{1}^{\left(k\right)},Z_{2}^{\left(k^{\prime }\right)})=\sqrt{\frac{r_{1}^{\left(k\right)}}{r_{1}^{\left(k\right)}+r_{1}^{\left(0\right)}}\frac{r_{1}^{\left(k^{\prime }\right)}}{r_{1}^{\left(k^{\prime }\right)}+r_{1}^{\left(0\right)}}}\sqrt{\frac{r_{1}^{\left(k\right)}r_{1}^{\left(0\right)}}{r_{1}^{\left(k\right)}+r_{1}^{\left(0\right)}}\frac{r_{2}^{\left(k\right)}+r_{2}^{\left(0\right)}}{r_{2}^{\left(k\right)}r_{2}^{\left(0\right)}}},k\neq k^{\prime },$ with $k,k^{\prime }\in \{1,2\}$.

We test the null hypotheses: $H_{01}:\{\theta^{\left(1\right)}\leq 0\},\;H_{02}:\{\theta^{\left(2\right)}\leq 0\}$ with the global null hypothesis denoted by $H_{0}:\{\theta^{\left(1\right)}=\theta^{\left(2\right)}=0\}$. Let $u_{j}^{\left(1\right)},l_{j}^{\left(1\right)}$ and $u_{j}^{\left(2\right)},l_{j}^{\left(2\right)}$ be the critical values at stage *j* for $T_{1}$ and $T_{2}$, respectively, used to test the hypotheses, with $u_{2}^{\left(k\right)}=l_{2}^{\left(k\right)},k\in \{1,2\}$.

The proposed design then takes into account the order among the treatment effects when making the decisions at the first stage and the final analysis and a set of decision rules consistent with this order is given in Table 1. For example, if both *Z*‐statistics cross the upper bounds at the interim analysis, the trial is stopped for efficacy (as in a conventional MAMS design). In contrast to the traditional MAMS design, the trial continues if there is contradicting evidence with respect to the order, for example, if $Z_{1}^{\left(2\right)}$ crosses the upper bound, but there is not enough evidence to claim superiority of $T_{1}$ to control, then both arms are continued to the next stage.

**TABLE 1:** Combination of the decision rules in the 3‐arm 2‐stage trial with $\theta^{\left(1\right)}\geq \theta^{\left(2\right)}$

	$Z_{j}^{\left(1\right)}\geq u_{j}^{\left(1\right)}$	$l_{j}^{\left(1\right)}<Z_{j}^{\left(1\right)}<u_{j}^{\left(1\right)}$	$Z_{j}^{\left(1\right)}\leq l_{j}^{\left(1\right)}$
$Z_{j}^{\left(2\right)}\geq u_{j}^{\left(2\right)}$	Stop: select $T_{1},T_{2}$	Proceed with *T*_1_, *T*_2_	Proceed with *T*_1_, *T*_2_
$l_{j}^{\left(2\right)}<Z_{j}^{\left(2\right)}<u_{j}^{\left(2\right)}$	Proceed with $T_{2}$	Proceed with $T_{1},T_{2}$	Drop both arms
$Z_{j}^{\left(2\right)}\leq l_{j}^{\left(2\right)}$	Stop: select $T_{1}$	Proceed with $T_{1}$	Drop both arms

*Note*: Cells colored in red correspond to contradicting evidence.

The idea behind these decision rules is that at any stage the effectiveness of $T_{2}$ can be claimed only if $T_{1}$ can be declared superior to the control. Therefore, $T_{1}$ can be regarded as a gatekeeper.^15^ Following this procedure, depending on the context, alternative decisions could be considered for the cells colored in red in Table 1 (see Section 2 of the Supporting Information for more discussion).

### 3.1 Family‐wise error rate

For confirmatory clinical trials, control of the family wise error rate (FWER) in the strong sense at level α, that is the probability to reject at least one true null hypothesis, is often required.^16^ Using the rules described in Table 1, the FWER for the 3‐arm 2‐stage ORD can be written as

(1) $$\begin{array}{ll} & P(\text{rejecting at least one true}\;H_{0k},\;k\in \{1,2\}\;|\;H_{0})=P\left(Z_{1}^{\left(1\right)}\geq u_{1}^{\left(1\right)}\;|\;H_{0}\right)+\\ & P\left(Z_{2}^{\left(1\right)}\geq u_{2}^{\left(1\right)},l_{1}^{\left(1\right)}<Z_{1}^{\left(1\right)}<u_{1}^{\left(1\right)}\;|\;H_{0}\right)+P\left(Z_{2}^{\left(1\right)}\geq u_{2}^{\left(1\right)},Z_{1}^{\left(1\right)}\leq l_{1}^{\left(1\right)},Z_{1}^{\left(2\right)}\geq u_{1}^{\left(2\right)}\;|\;H_{0}\right). \end{array}$$

Equation (1) shows that the events used for the computation of the type I error under the global null hypothesis ($\left\{\text{Reject}\;H_{01}\;\text{and}\;H_{02}\},\{\text{Reject}\;H_{01}\;\text{not}\;H_{02}\right\}$) are a subset of the events ($\left\{\text{Reject}\;H_{01}\cup H_{02}\right\}$) used in the MAMS design of Magirr et al.^3^ Thus, the probability of rejecting at least one hypothesis under the global null will be smaller for the ORD compared to the MAMS design, while the probability of rejecting neither hypothesis will be smaller for MAMS if the same bounds are used. It is worth noting that overall, the critical bounds, if these are the same for all active treatments $u_{1}^{\left(1\right)}=u_{1}^{\left(2\right)}=u_{1}$, $u_{2}^{\left(1\right)}=u_{2}^{\left(2\right)}=l_{2}^{\left(1\right)}=l_{2}^{\left(2\right)}=u_{2}$, $l_{1}^{\left(1\right)}=l_{1}^{\left(2\right)}=l_{1}$, for the 3‐arm 2‐stage ORD are smaller in each stage compared to the MAMS design of Magirr et al^3^ (see Section 7 of the Supporting Information). Consequently the ORD design is strictly more powerful than the MAMS design under these assumptions.

The critical bounds for the given treatment arm can be defined as function of a (possibly arm‐specific) parameter, that is, $u_{j}^{\left(k\right)}=u_{j}^{\left(k\right)}(a^{\left(k\right)}),l_{j}^{\left(k\right)}=l_{j}^{\left(k\right)}(a^{\left(k\right)}),j\in \{1,2\},k\in \{1,2\}$, which can be searched over a grid of values for $a^{\left(k\right)}$ in order to strongly control the FWER at level α. If $a^{\left(1\right)}=a^{\left(2\right)}=a$, then a unique solution can be found restricting the search over *a* such that the expression given in Equation (1) is below α under the global null hypothesis. In this case, the solution is unique either when the search is based on different boundary shapes $u_{j}^{\left(k\right)},l_{j}^{\left(k\right)}$ or when the same boundary shapes are used for all experimental arms—$u_{j}^{\left(k\right)}=u_{j},l_{j}^{\left(k\right)}=l_{j}$ for all k∈{1,2}. If $a^{\left(k\right)}$ are not the same for each arm, additional constraints are required for the uniqueness of the solution and to maintain the strong control of the FWER at level α, such as the control under the partial null hypotheses. In the 3‐arm setting, for example, this is $\left(\theta^{\left(1\right)},0\right)$. However, different values of $\theta^{\left(1\right)}$ can provide different boundaries, and so the solution is unique for the specific value of $\theta^{\left(1\right)}$ (see Section 7 for more details).

Theorem 1 below then shows that, if the same bounds, $\left(u_{j},l_{j}),j\in \{1,2\right\}$ are used for each arm, and the same allocation ratios (with respect to the control) are used for all active treatments, the FWER is maximized under the global null hypothesis and hence the above ensures strong control of the FWER.

Theorem 1 *Consider a 3‐arm 2‐stage ORD design and denote the global null hypothesis by* $H_{0}:\theta^{\left(1\right)}=\theta^{\left(2\right)}=0$ *. Let* $u_{1}^{\left(1\right)}=u_{1}^{\left(2\right)}=u_{1}$ *,* $u_{2}^{\left(1\right)}=u_{2}^{\left(2\right)}=l_{2}^{\left(1\right)}=l_{2}^{\left(2\right)}=u_{2}$ *,* $l_{1}^{\left(1\right)}=l_{1}^{\left(2\right)}=l_{1}$ *be the critical bounds such that Equation (* *1* *) is below* α *under the global null hypothesis. Let us assume that there are equal numbers of patients on each active treatment within each stage*: $r_{j}^{\left(k\right)}=r_{j},\;\forall k\in \{1,2\}$.

*Let* $\theta_{0}$ *be a vector where at least one treatment effect is less or equal to 0. Then,*

$$\begin{array}{llll} & P(\text{rejecting at least one true}\;H_{0k},k\in \{1,2\}\;|\;\theta_{0})\leq \; & & \\ & P(\text{rejecting at least one true}\;H_{0k},k\in \{1,2\}\;|\;H_{0})\leq \alpha \; & & \end{array}$$

The proofs of all theorems are given in Section 1 of the Supporting Information.

### 3.2 Power requirement

To power the study, we consider the configuration $\theta =(\theta^{\left(1\right)},\theta^{\left(2\right)})$, where $\theta^{\left(1\right)}\geq \theta^{\left(2\right)}\geq \delta_{0}>0$ and $\delta_{0}$ is the minimum clinically relevant difference. The ORD is then powered at (1−β) to reject both hypotheses under $\theta =(\theta^{\left(1\right)},\theta^{\left(2\right)})$, $\theta^{\left(1\right)}\geq \theta^{\left(2\right)}\geq \delta_{0}>0$ when Equation (2) is satisfied:

(2) $$\begin{array}{ll} & P\left(Z_{1}^{\left(1\right)}\geq u_{1}^{\left(1\right)},Z_{1}^{\left(2\right)}\geq u_{1}^{\left(2\right)}\;|\;\theta \right)+\\ & P\left(Z_{2}^{\left(1\right)}\geq u_{2}^{\left(1\right)},Z_{2}^{\left(2\right)}\geq u_{2}^{\left(2\right)},l_{1}^{\left(1\right)}<Z_{1}^{\left(1\right)}<u_{1}^{\left(1\right)},Z_{1}^{\left(2\right)}\geq u_{1}^{\left(2\right)}\;|\;\theta \right)+\\ & P\left(Z_{2}^{\left(1\right)}\geq u_{2}^{\left(1\right)},Z_{2}^{\left(2\right)}\geq u_{2}^{\left(2\right)},Z_{1}^{\left(1\right)}\leq l_{1}^{\left(1\right)},Z_{1}^{\left(2\right)}\geq u_{1}^{\left(2\right)}\;|\;\theta \right)+\\ & P\left(Z_{2}^{\left(2\right)}\geq u_{2}^{\left(2\right)},Z_{1}^{\left(1\right)}\geq u_{1}^{\left(1\right)},l_{1}^{\left(2\right)}<Z_{1}^{\left(2\right)}<u_{1}^{\left(2\right)}\;|\;\theta \right)+\\ & P\left(Z_{2}^{\left(1\right)}\geq u_{2}^{\left(1\right)},Z_{2}^{\left(2\right)}\geq u_{2}^{\left(2\right)},l_{1}^{\left(1\right)}<Z_{1}^{\left(1\right)}<u_{1}^{\left(1\right)},l_{1}^{\left(2\right)}<Z_{1}^{\left(2\right)}<u_{1}^{\left(2\right)}\;|\;\theta \right)\geq 1-\beta . \end{array}$$

Theoretical considerations and numerical evaluations have shown that, if Pocock boundaries^17^ for both treatments are used, $u_{1}^{\left(1\right)}=u_{1}^{\left(2\right)}=u_{1},\;l_{1}^{\left(1\right)}=l_{1}^{\left(2\right)}=-u_{1}$, $u_{2}^{\left(1\right)}=u_{2}^{\left(2\right)}=u_{1}$, and critical values found such that Equation (1) is below α, the power of the ORD design is, practically, no smaller than a fixed balanced sample design with the same sample size. Furthermore, for a number of treatment effects, it was found to be strictly positive. Full details of these considerations are given in Section 3 of the Supporting Information.

## 4 GENERALIZATION OF THE ORD TO *K*‐arm *J*‐stage

Consider a clinical trial with K−1 active treatment arms, $T_{1},\ldots ,T_{K-1}$, against a control treatment and *J* stages and denote the treatment effect comparing treatment *k* against control by $\theta^{\left(k\right)}$. We denote the vector of treatment effects by $\theta =(\theta^{\left(1\right)},\theta^{\left(2\right)},\ldots ,\theta^{\left(K-1\right)})$. The null hypotheses of interest are $H_{01}:\{\theta^{\left(1\right)}\leq 0\}\;,\ldots ,\;H_{0K-1}:\{\theta^{\left(K-1\right)}\leq 0\}$. Let $Z_{j}^{\left(k\right)}$ denote the test statistic based on all data up to stage *j* for comparison k∈{1,…,K−1} as before and assume that the following order relationship holds: $\theta^{\left(1\right)}\geq \theta^{\left(2\right)}\geq \cdots \geq \theta^{\left(K-1\right)}$. Let $u_{j}^{\left(k\right)},l_{j}^{\left(k\right)},k\in \{1,\ldots ,K-1\},j\in \{1,\ldots ,J\}$ be the critical values at stage *j* with $u_{J}^{\left(k\right)}=l_{J}^{\left(k\right)},k\in \{1,\ldots ,K-1\}$.

The decision rules at the interim analyses follow the same principle as for the 3‐arm 2‐stage design defined above. The decisions are made in order to be able to select all promising treatment arms at the end of the trial and $H_{0k}$ can only be rejected if all $H_{0k^{\prime }}$, $k^{\prime }<k$ have been rejected. Once $H_{0k}$ has been rejected, the recruitment to arms $T_{{ℒ}_{j}},\ldots ,T_{k}$ is stopped, where ${ℒ}_{j}$ is the lowest index of a treatment arms remaining in the trial at stage *j*. If there is contradicting evidence with respect to the order at stage *j*, that is when $Z_{j}^{\left(k\right)}\geq u_{j}^{\left(k\right)}$ and there is at least one $k^{\prime }<k$ such that $Z_{j}^{\left(k^{\prime }\right)}<u_{j}^{\left(k^{\prime }\right)}$, then recruitment to these arms continue. As for the 3‐arm 2‐stage design, if there is sufficient evidence to drop arm *k*, that is when $Z_{j}^{\left(k\right)}\leq l_{j}^{\left(k\right)}$, and if there is any contradicting evidence for $k^{\prime }>k$ then the recruitment to arms $T_{k},\ldots ,T_{{ℋ}_{j}}$ is stopped, where ${ℋ}_{j}$ is the highest index on the treatment arms remaining in the trial at stage *j*. A general algorithm for the decision‐making in this setting is given in Algorithm 1.

Algorithm 1 **Rules for *K*‐arm *J*‐stage ORD when $\theta^{\left(1\right)}\geq \theta^{\left(2\right)}\geq \cdots \geq \theta^{\left(K-1\right)}$
**

1

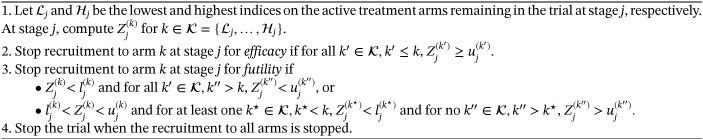

Let $M_{j}$ be a random variable representing the number of arms (including the control) at stage *j* when $H_{01}$ failed to be rejected at stage j−1. Because of the hierarchy in testing the hypotheses, the FWER for an *K*‐arm *J*‐stage design can be written as

(3) $$\begin{array}{ll} & P(\text{rejecting at least one true}\;H_{0k},\;k\in \{1,\ldots ,K-1\}\;|\;H_{0})=\\ & \overset{J}{\underset{j=1}{\sum }}P(\text{rejecting}\;H_{01}\;\text{at}\;j\text{th stage},H_{01}\;\text{not rejected at stage}\;s,\forall s<j\;|\;H_{0})=\\ & P\left(Z_{1}^{\left(1\right)}\geq u_{1}^{\left(1\right)}\;|\;H_{0}\right)+\overset{J}{\underset{j=2}{\sum }}\overset{K}{\underset{m=2}{\sum }}P(Z_{j}^{\left(1\right)}\geq u_{j}^{\left(1\right)}\;|\;M_{j}=m,H_{0})\times P(M_{j}=m), \end{array}$$

where $P\left(M_{j}=m\right)$ is the probability that at the previous stage $H_{01}$ failed to be rejected and the number of arms were at least *m*. One can show that the following iterative equality holds

$$P(M_{j}=m)=\overset{K}{\underset{c=m}{\sum }}P(A_{j,c-m+1}^{\left(c-1\right)}\;|\;M_{j-1}=c,H_{0})\times P(M_{j-1}=c),$$

with at the first stage $P(M_{1}=K)=1$ and 0 otherwise. The set $A_{j,c-m+1}^{\left(c-1\right)}$ defines the event that $H_{01}$ failed to be rejected at stage j−1 when the number of treatment arms (including the control arm) in the trial were *c*. This set is formally defined in Table 2. In the definition of $A_{j,c-m+1}^{\left(c-1\right)}$, the superscript (c−1) indicates the number of active treatment arms that are still in the trial at stage j−1, while the subscript c−m+1 refers to the number of active treatment arms (that is equal to c−m) that have been dropped before reaching the stage *j*.

**TABLE 2:** Sets of events for K‐arm J‐stage design with k∈{1,…,K−1}

Set	Definition
$C_{j}^{\left(k\right)}$	$\left\{l_{j-1}^{\left(k\right)}<Z_{j-1}^{\left(k\right)}<u_{j-1}^{\left(k\right)}\right\}$
$S_{j}^{\left(k\right)}$	$\left\{Z_{j-1}^{\left(k\right)}\leq l_{j-1}^{\left(k\right)}\right\}$
$E_{j}^{\left(k\right)}$	$\left\{\left((C_{j}^{\left(1\right)}\cup S_{j}^{\left(1\right)})\cap \bar{\left(C_{j}^{\left(k\right)}\cup S_{j}^{\left(k\right)}\right)}\right)\cup (E_{j}^{\left(k-1\right)}\cap C_{j}^{\left(k\right)})\right\}$
$E_{j}^{\left(0\right)}$	Ω
$A_{j,1}^{\left(k\right)},k>0$	$E_{j}^{\left(k\right)}$
$A_{j,2}^{\left(k\right)},k>1$	$E_{j}^{\left(k-1\right)}\cap S_{j}^{\left(k\right)}$
$A_{j,k+1-s}^{\left(k\right)},k>2,s=1:k-2$	$E_{j}^{\left(s\right)}\cap {S_{j}^{\left(s+1\right)}⋂}_{t=s+2}^{k}(C_{j}^{\left(t\right)}\cup S_{j}^{\left(t\right)})$

*Note*: Ω is the whole sample space.

While the expression for the FWER in the general case is cumbersome, for a fixed number of stages (arms), the FWER for *K*‐arm (*J*‐stage) can be found iteratively—an example for 2‐stage trials is given in Section 4 of the Supporting Information.

The critical bounds for a *K*‐arm *J*‐stage ORD can be, again, defined as functions of parameters $a^{\left(k\right)}$ such that $u_{j}^{\left(k\right)}=u_{j}^{\left(k\right)}(a^{\left(k\right)}),l_{j}^{\left(k\right)}=l_{j}^{\left(k\right)}(a^{\left(k\right)}),j\in \{1,\ldots ,J\},k\in \{1,\ldots ,K-1\}$. Thus, as for the 3‐arm 2‐stage setting, under the constraint of $a^{\left(1\right)}=a^{\left(2\right)}=\cdots =a^{\left(K-1\right)}=a$, a unique solution can be found to control the FWER at level α in the strong sense. Theorem 2 below then shows that, if the same bounds, $\left(u_{j},l_{j}),j\in \{1,\ldots ,J\right\}$ are used for each arm and the same allocation ratios (with respect to the control) are used for all active treatments, the FWER is maximized under the global null hypothesis $H_{0}:\{\theta^{\left(1\right)}=\theta^{\left(2\right)}=\cdots =\theta^{\left(K-1\right)}=0\}$ and hence the above ensures strong control of the FWER.

Theorem 2 *Consider a K‐arm J‐stage ORD design and denote the global null hypothesis by* $H_{0}:\{\theta^{\left(1\right)}=\theta^{\left(2\right)}=\cdots =\theta^{\left(K-1\right)}=0\}$ *. Let* $u_{j}^{\left(k\right)}=u_{j}$ *,* $l_{j}^{\left(k\right)}=l_{j},u_{J}^{\left(k\right)}=l_{J}^{\left(k\right)}=u_{J},k\in \{1,\ldots ,K-1\}$ *be the critical values such that Equation (* *3* *) is below* α *under the global null hypothesis. Assume that there are equal numbers of patients on each active treatment within each stage*: $r_{j}^{\left(k\right)}=r_{j},\;\forall k\in \{1,\ldots ,K-1\}$ *. Let* $\theta_{0}$ *be the vector where at least one treatment effect is less or equal to 0*.

*Then,*

$$\begin{array}{llll} & P(\text{rejecting at least one true}\;H_{0k},\;k\in \{1,\ldots ,K-1\}\;|\;\theta_{0})\leq \; & & \\ & P(\text{rejecting at least one true}\;H_{0k},\;k\in \{1,\ldots ,K-1\}\;|\;H_{0})\leq \alpha \; & & \end{array}$$

In the next session, a simulation study will be described in order to apply the proposed design in the context of the asthma trial.^14^

## 5 CASE STUDY

### 5.1 Setting

We revisit the results of the clinical trial of *Tiotropium add‐on therapy in adolescents with moderate asthma: A 1‐year randomized controlled trial* (NCT01257230).^14^ Patients were randomized in a 1:1:1 ratio to receive 5μg or 2.5μg of once‐daily tiotropium or placebo. The null hypotheses were tested in a stepwise manner to control the type I error at level α=0.025. The study was powered at 80% to detect a difference of 120 mL between treatments in the change from baseline of peak FEV${}_{1[0-3h]}$ assuming a common SD of 340 mL. It was found that 127 patients per group were needed, resulting in a maximum sample size of 381 patients. The trial is revisited using the ORD, which can be applied assuming a monotonic dose‐response relationship.

In line with the original trial we assume that the change from baseline of peak FEV${}_{1[0-3h]}$ is normally distributed with SD σ=340,k∈{0,1,2}, and common baseline mean ${\text{FEV}}_{1}$ of $\mu^{\left(0\right)}=2747$. As in the original study, we consider an improvement of ${\text{FEV}}_{1}$ of 120 of interest and hence consider the following values for $\theta^{\left(k\right)},\;k\in \{1,2\}$: θ=(0,0),θ=(120,0) and θ=(120120). The design is powered at 80% to reject all hypotheses or at least one hypothesis (in order to compare the sample sizes between the ORD and the original trial design) when all doses have the same effect compared to the placebo. Additionally to the achieved power, the efficiency of the proposed design is measured by its expected sample size (ESS), that is the mean number of patients recruited to the trial before it is terminated.

We consider one‐ and two‐stage ORD designs and note that the one‐stage ORD design corresponds to the hierarchical testing strategy used in the original design. For the two‐stage design the interim analysis takes place after half of the total sample size has been observed and triangular critical bounds^18^ are used. The numerical results found using R^19^ and $10^{6}$ replicate simulations.

### 5.2 Numerical results

Consider the 3‐arm 1‐stage ORD using the maximum total sample size of 381 patients that corresponds to the same maximum total sample size originally planned for the study. In this setting, the critical bound at the final analysis is $u_{1}=z_{1-\alpha }=z_{0.975}=1.96$. Table 3 describes the results of the simulation.

**TABLE 3:** Results of the simulations that revisit the NCT01257230 trial using the ORD

Design powered to reject all hypotheses							
$\theta^{\left(1\right)}$	$\theta^{\left(2\right)}$	Max. SS	Stages	Reject all	Reject $H_{01}$ not $H_{02}$	Reject at least one $H_{0k}$	ESS
0	0	474	1	0.005	0.020	0.025	474.00
		534	2	0.004	0.021	0.025	316.39
120	0	474	1	0.025	0.856	0.881	474.00
		534	2	0.025	0.854	0.879	371.83
120	120	474	1	**0.803**	0.078	0.881	474.00
		534	2	**0.802**	0.081	0.883	399.81

Design powered to reject at least one hypothesis							
$\theta^{\left(1\right)}$	$\theta^{\left(2\right)}$	Max. SS	Stages	Reject all	Reject $H_{01}$ not $H_{02}$	Reject at least one $H_{0k}$	ESS
0	0	381	1	0.005	0.021	0.025	381.00
		426	2	0.004	0.021	0.025	252.43
120	0	381	1	0.025	**0.779**	**0.803**	381.00
		426	2	0.024	0.774	0.798	304.67
120	120	381	1	**0.691**	0.112	**0.803**	381.00
		426	2	0.684	0.117	**0.802**	331.89

*Note*: For the two‐stage design, the triangular bounds are used. Proportions refer to $10^{6}$ replications and values of interest are in bold.

Abbreviations: ESS, expected sample size; Max. SS, maximum sample size.

It can be seen that the FWER is controlled at level α=0.025 under all considered null scenarios. For the scenarios where there is at least one dose that is superior to control, the probability to reject at least one hypothesis is 80% as required in the original study. Note that 80% is the probability of rejecting at least one dose considering any rejections and not only correct rejections. Therefore, when no interim analyses are planned, the ORD requires the same maximum sample size as in the original study if it is powered to reject at least one hypothesis. It is worth noting that the probability of rejecting all hypotheses, if all of them are true, is around 69%.

The distinguishing feature of the proposed ORD is that it allows to include interim analyses during the trial. Table 3 shows the operating characteristics of the design when an interim analysis takes place after observing half of the maximum sample sizes. If the study is powered to reject at least one hypothesis at 80%, a maximum sample size of 426 patients is needed—45 more patients than for the 1‐stage design. The gain from using a two‐stage design arises in terms of the expected sample size—on average the number of patients is expected to be below 332 under each scenario. At the same time, under this power configuration, the probability to identify the smallest promising dose is at 68% similar to the single‐stage design.

Furthermore, it is argued by the construction of the ORD proposed in Section 3 that if it is of interest to identify the lowest dose with the promising treatment effect, the trial should be powered to reject all hypotheses. In order to identify the lowest effective dose with the desirable 80% probability, a single‐stage trial would require 474 patients and the two‐stage design 534 patients. As before, the inclusion of an interim analysis and the use of triangular bounds lead to the reduction of the expected number of patients. Specifically, on average the number of patients is expected to be below 400 under each scenario.

Overall, the ORD was found to reproduce the sample size calculation of the original study when no interim analyses were planned and is powered to reject at least one hypothesis. At the same time, the ORD allows the inclusion of interim analyses during the trial. When an interim analysis is planned during the trial, the ORD has shown to be an efficient design as it allows to stop the trial earlier or to drop unpromising doses with high probability before the end of the study.

The results discussed in this section use triangular bounds. Qualitatively similar results using Pocock^17^ and O'Brien and Fleming^20^ boundaries are provided in Section 6 of the Supporting Information. To further investigate the design characteristics of the proposed ORD, an extensive simulation study is conducted in Section 6.

## 6 NUMERICAL EVALUATIONS

### 6.1 Setting

As in the motivating example, consider a clinical trial setting with 3 treatments arms, 2 experimental and a control with 1:1:1 allocation ratio. Consider a single‐stage design (that is a fixed sample design (FSD) with hierarchical testing, FSD(h)) and a two‐stage design with one pre‐planned interim analysis at the middle of the trial. The objective of the trial is to find all promising treatment arms. The FWER is to be controlled below α=0.05 and the power of trial is to be at least 80% to reject both hypotheses when both treatment arms have the same effect compared to the control. The patients' responses on treatment arm *k* are assumed to have normal distribution with mean $\mu^{\left(k\right)}$ and SD σ=1. For the control group $\mu^{\left(0\right)}=1$, while for the treatment arms $\mu^{\left(k\right)}=\mu^{\left(0\right)}+\theta^{\left(k\right)}$. We fix the clinically relevant difference to be 0.5. Therefore, the scenario under which the ORD is powered is θ=(0.5,0.5). We evaluate the performance of the design under various treatment effects configurations when the treatment effect on the first arm is fixed to be 0.5 and the treatment effect on the second arm is varied: $\theta =(0.5,\theta^{\left(2\right)}),\;\theta^{\left(1\right)}\geq \theta^{\left(2\right)}$, and $\theta^{\left(2\right)}\in \{0,0.1,0.2,0.3,0.4,0.5\}$. For the 3‐arm 2‐stage ORD, the bounds are found under the conditions $a^{\left(k\right)}=a$ (ie, the same for each treatment arm) and they are found using a grid search—based on the triangular boundary shape^18^ for the all experimental treatments—over one single parameter. Thus, the solution found is unique.

### 6.2 Competing approaches

The proposed ORD is compared to the FSD,^21^ in which the total sample size is specified at the design stage of the trial and it is not subject to adaptations during the process of the trial. The hypotheses are tested at the end of the trial only and any hypothesis can be rejected independently of the other.

The second comparator is the MAMS design by Magirr et al.^3^ However, the conventional MAMS design is not an appropriate comparator as it does stop as soon as at least one hypothesis is rejected. Therefore, the following modification of the MAMS design is considered for the comparison. At the interim analysis, if a *Z*‐statistic corresponding to one arm crosses the upper or lower bound while another does not, the trial will still continue with the treatment that did not cross a bound. The design is referred to as the MAMS(m).

The FWER expression for MAMS(m) is the same as derived by Magirr et al^3^ but the power expression changes. To power a 3‐arm 2‐stage design, for a given configuration of θ and a pre‐specified level β, we search for the sample size that satisfies

(4) $$\begin{array}{ll} & P\left(Z_{1}^{\left(1\right)}\geq u_{1}^{\left(1\right)},Z_{1}^{\left(2\right)}\geq u_{1}^{\left(2\right)}\;|\;\theta \right)+\\ & P\left(Z_{2}^{\left(1\right)}\geq u_{2}^{\left(1\right)},Z_{2}^{\left(2\right)}\geq u_{2}^{\left(2\right)},l_{1}^{\left(1\right)}<Z_{1}^{\left(1\right)}<u_{1}^{\left(1\right)},l_{1}^{\left(2\right)}<Z_{1}^{\left(2\right)}<u_{1}^{\left(2\right)}\;|\;\theta \right)+\\ & P\left(Z_{2}^{\left(1\right)}\geq u_{2}^{\left(1\right)},l_{1}^{\left(1\right)}<Z_{1}^{\left(1\right)}<u_{1}^{\left(1\right)},Z_{1}^{\left(2\right)}\geq u_{1}^{\left(2\right)}\;|\;\theta \right)+\\ & P\left(Z_{2}^{\left(2\right)}\geq u_{2}^{\left(2\right)},l_{1}^{\left(2\right)}<Z_{1}^{\left(2\right)}<u_{1}^{\left(2\right)},Z_{1}^{\left(1\right)}\geq u_{1}^{\left(1\right)}\;|\;\theta \right)\geq 1-\beta . \end{array}$$

### 6.3 Numerical results

Two main simulation studies are conducted to compare the three competing approaches. In the first one, each design is constructed such that it yields 80% power to reject both null hypotheses under the alternative hypothesis. The second one compares the power between approaches based on the common sample size.

#### 6.3.1 Same power requirement for all designs

In this subsection, the results when all design are powered at 80% to reject both null hypotheses are provided. The resulting design specifications and operating characteristics under the global null hypothesis are provided in Table 4.

**TABLE 4:** FWER, maximum sample sizes (Max. SS), expected sample sizes (ESS), and critical bounds under θ=(0,0) for the 3‐arm 2‐stage ORD, 3‐arm 1‐stage ORD (FSD(h)), FSD, and MAMS(m) designs when all designs are powered at 80% to reject both hypotheses under θ=(0.5,0.5)

Design	$u_{1},u_{2},l_{1}$	Max. SS	ESS	Reject at least one $H_{0k}$
FSD	1.917, ‐, 1.917	231	231.0	0.05
FSD(h)	1.644, ‐, 1.644	192	192.0	0.05
ORD	1.898, 1.789, 0.633	222	134.4	0.05
MAMS(m)	2.179, 2.055, 0.726	264	166.6	0.05

*Note*: 3‐arm 2‐stage ORD and MAMS(m) use triangular bounds. Results are provided using $10^{6}$ replications.

Under θ=(0,0), all designs control the type I error at level α=0.05 as expected. The total maximum sample sizes necessary to reach a power of 80% is 231 for the FSD, 192 for the FSD with a hierarchical test (FSD(h)) which is akin to a single‐stage ORD design, 222 for the 3‐arm 2‐stage ORD and 264 for the MAMS(m) designs. The maximum sample size (to achieve the same power to reject both hypotheses) for the FSD is greater compared to FSD(h) and the two‐stage ORD as it does not account for the hierarchy in testing.

**FIGURE 1:**
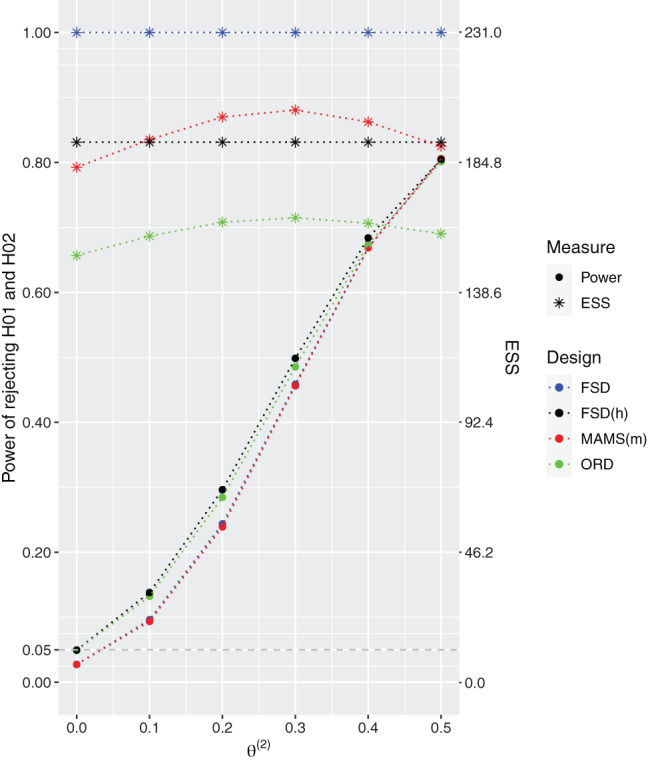
Power and expected sample sizes (ESS) under $\theta =(0.5,\theta^{\left(2\right)})$ and $\theta^{\left(2\right)}\in \{0,0.1,0.2,0.3,0.4,0.5\}$ for the FSD, 3‐arm 1‐stage ORD (FSD(h)), 3‐arm 2‐stage ORD, and MAMS(m) designs when all designs are powered at 80% to reject both hypotheses under θ=(0.5,0.5). 3‐arm 2‐stage ORD and MAMS(m) use triangular bounds. Results are provided using $10^{6}$ replications

The designs' performances under the configuration $\theta^{\left(1\right)}\geq \theta^{\left(2\right)}$ and $\theta^{\left(2\right)}\in \{0,0.1,0.2,0.3,0.4,0.5\}$ are presented in Figure 1. When the second treatment arm is no different to control, $\theta^{\left(2\right)}=0$, the probability of rejecting the null hypothesis for this arm is controlled at α=0.05 for all designs and when θ=(0.5,0.5) all the designs satisfy the power requirement at 80%. However, under all other considered non‐zero values of $\theta^{\left(2\right)}$, the probability of rejecting both hypotheses is higher for the approaches accounting for the hierarchy, FSD(h) and ORD, than for other competing designs. The gain from using a two‐stage ORD design compared to FSD(h) arises in terms of expected sample sizes which is strictly lower for the two‐stage design. The 3‐arm 2‐stage ORD has noticeably lower expected sample size (ESS) compared to all other designs ranging from 13% to 35% depending on the simulation scenario. The largest difference in power (an increase of around 5%) between the 3‐arm 2‐stage ORD and the MAMS(m) design is achieved under $\theta^{\left(2\right)}=0.2$.

#### 6.3.2 Common sample size for all designs

In this subsection, the three designs are compared with a common maximum sample size of 222 patients, which is the maximum sample size necessary for the 2‐stage ORD in order to reject both hypotheses at 80% under θ=(0.5,0.5).

**FIGURE 2:**
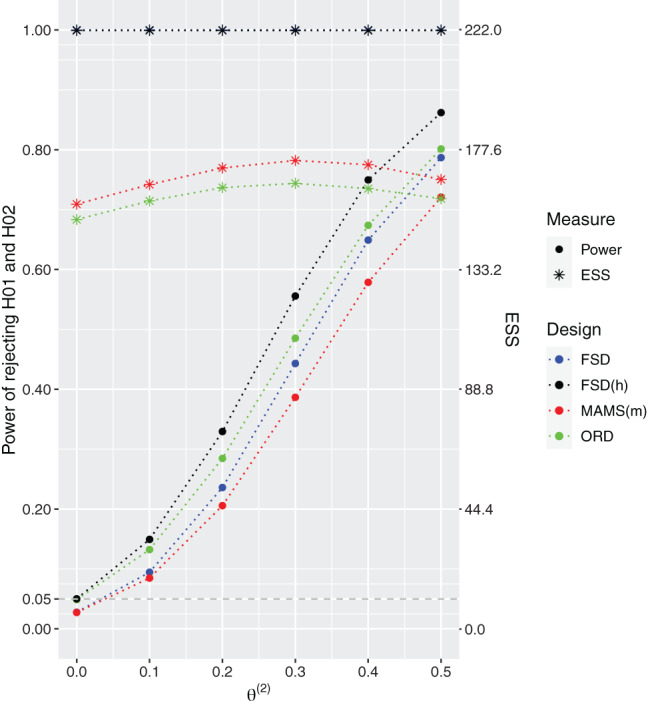
Power and expected sample sizes (ESS) under $\theta =(0.5,\theta^{\left(2\right)})$ and $\theta^{\left(2\right)}\in \{0,0.1,0.2,0.3,0.4,0.5\}$ for the FSD, 3‐arm 1‐stage ORD (FSD(h)), 3‐arm 2‐stage ORD, and MAMS(m) designs when all designs have the same common total sample size equal to 222 patients. 3‐arm 2‐stage ORD and MAMS(m) use triangular bounds. Results are provided using $10^{6}$ replications

The design specifications and operating characteristics of the designs under the global null hypothesis are provided in Table 5, while the designs' performances under the configuration $\theta^{\left(1\right)}\geq \theta^{\left(2\right)}$ and $\theta^{\left(2\right)}\in \{0,0.1,0.2,0.3,0.4,0.5\}$ are presented in Figure 2.

**TABLE 5:** FWER, maximum sample sizes (Max. SS), expected sample sizes (ESS), and critical bounds under θ=(0,0) for the 3‐arm 2‐stage ORD, 3‐arm 1‐stage ORD (FSD(h)), FSD, and MAMS(m) designs when all designs have the same common total sample size equal to 222 patients

Design	$u_{1},u_{2},l_{1}$	Max. SS	ESS	Reject at least one $H_{0k}$
FSD	1.917, ‐, 1.917	222	222.0	0.05
FSD(h)	1.644, ‐, 1.644	222	222.0	0.05
ORD	1.898, 1.789, 0.633	222	134.4	0.05
MAMS(m)	2.179, 2.055, 0.726	222	140.1	0.05

*Note*: 3‐arm 2‐stage ORD, and MAMS(m) use triangular bounds. Results are provided using $10^{6}$ replications.

As expected all designs control the FWER under the global null. Under the scenario when the second treatment arm is no different to control, $\theta^{\left(2\right)}=0$, the probability of rejecting the null hypothesis for this arm is controlled at α=0.05. Under all other considered non‐zero values of $\theta^{\left(2\right)}$, the approaches accounting for the hierarchy, FSD(h) and ORD, result in higher power compared to the other competing designs. The gain from using a two‐stage ORD design compared to FSD(h) can once more be seen in terms of expected sample sizes which is strictly lower for the two‐stage design. The 2‐stage ORD has lower ESS compared to the other designs with reductions between 3% and 32%. The largest difference in power—around 9.8%—between the ORD and the MAMS(m) design is achieved under $\theta^{\left(2\right)}=0.3$.

Overall, the ORD results in noticeable gains across all considered scenarios both in terms of power and expected sample size. Therefore, the inclusion of the order restriction into the decision rules for the decision‐making can provide advantages in power and/or expected sample size compared to standard approach to multi‐arm trials, specifically, the FSD and the MAMS(m).

## 7 DIFFERENT BOUNDS FOR EACH TREATMENT ARM

In the results above the same bounds are used for both treatments. However, the proposed design allows for different boundary shapes to be used for different treatments which could lead to potential benefit in terms of power. In this section, we explore the effect of various boundary shapes on the operating characteristics of the designs.

We consider the setting as in Section 6.1 and let $u_{j}^{\left(1\right)}=u_{j}^{\left(1\right)}(a^{\left(1\right)}),l_{j}^{\left(1\right)}=l_{j}^{\left(1\right)}(a^{\left(1\right)})$ be the upper and lower bounds for $T_{1}$ at the stage *j*, and $u_{j}^{\left(2\right)}=u_{j}^{\left(2\right)}(a^{\left(2\right)}),l_{j}^{\left(2\right)}=l_{j}^{\left(2\right)}(a^{\left(2\right)})$ be the boundaries for $T_{2}$ at the stage *j*, being functions of $a^{\left(1\right)}$ and $a^{\left(2\right)}$, respectively. The critical bounds and the sample size could be searched over a grid of values for $a^{\left(1\right)}$ and $a^{\left(2\right)}$ in order to strongly control the FWER at level α and to satisfy the power requirements in Equation (2). To maintain strong control of the FWER at level α it is necessary to control the type I error when $\theta_{0}=(\theta^{\left(1\right)},0)$. Indeed, as shown in Section 1 of the Supporting Information, under $\theta_{0}=(\theta^{\left(1\right)},0)$ it holds

$$\begin{array}{llll} & P(\text{rejecting at least one true}\;H_{0k},\;k\in \{1,2\}\;|\;\theta_{0})=P(\text{reject}\;H_{02}\;|\;\theta_{0})= & & \\ & P(\text{reject}\;H_{02}\;|\;\text{reject}\;H_{01},\theta_{0})\times P(\text{reject}\;H_{01}\;|\;\theta_{0})\leq P(\text{reject}\;H_{02}\;|\;\text{reject}\;H_{01},\tilde{\theta_{0}}=(\infty ,0)),\; & & \end{array}$$

where $P(\text{reject}\;H_{02}\;|\;\text{reject}\;H_{01},\tilde{\theta_{0}}=(\infty ,0))$ tends to

$$P\left(Z_{1}^{\left(2\right)}>u_{1}^{\left(2\right)}\;|\;H_{02}\right)+P\left(Z_{2}^{\left(2\right)}>u_{2}^{\left(2\right)},l_{1}^{\left(2\right)}<Z_{1}^{\left(2\right)}<u_{1}^{\left(2\right)}\;|\;H_{02}\right).$$

Thus, the bounds for the second arm are searched over a grid of values of $a^{\left(2\right)}$ as for a 2‐arm design with 2 stages^22^ and then the bounds for $T_{1}$ are searched in order to satisfy Equation (1) under the null hypothesis. Finally, the sample size is searched to satisfy the power requirements, assuming equal allocation to all arms.

Several combinations of boundary shapes are compared considering all possibilities with constant POC,^17^ O'Brien and Fleming OBF^20^ and triangular TRIAN^18^ bounds. Among all these nine combinations of bounds, a subset of six is selected. For each shape of the bounds for $T_{1}$, two combinations are selected. These are those that provide the smallest ESS and the highest power compared to the other ones. The combinations that are excluded are the ones that use constant bounds for $T_{1}$ and $T_{2}$, the combination with O'Brien and Fleming and constant bounds for $T_{1}$ and $T_{2}$ respectively and the combination with triangular and constant bounds for $T_{1}$ and $T_{2}$ respectively. The complete set of results is provided in Section 5 of the Supporting Information.

**FIGURE 3:**
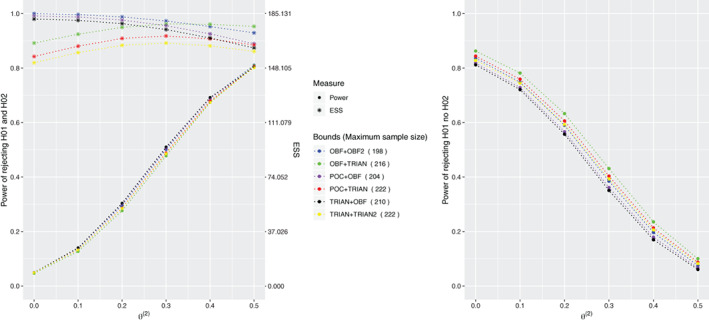
Probability of rejecting both hypotheses (left) and probability of rejecting the first but not the second hypothesis (right) under $\theta =(0.5,\theta^{\left(2\right)})$ and $\theta^{\left(2\right)}\in \{0,0.1,0.2,0.3,0.4,0.5\}$ for the 3‐arm 2‐stage ORD design when it is powered at 80% to reject both hypotheses under θ=(0.5,0.5). ORD uses the selected combination of bounds which control the type I error under θ=(∞,0). Results are provided using $10^{6}$ replications

The summary of operating characteristics, probability to reject both and probability to reject only one, and the expected sample size, for the proposed design using the six remaining combinations of boundary shapes is provided in Figure 3.

The design resulting in the highest power in rejecting both hypotheses among the subset of selected bounds is the one that uses triangular bounds for the treatment associated to the highest effect and O'Brien and Fleming bounds for the arm associated to the lowest treatment effect. Indeed, in the way that O'Brien and Fleming bounds are constructed, if $u_{1}^{\left(2\right)}>u_{1}^{\left(1\right)}$, then the trial tends to stop later and the final decision on $T_{2}$ is based on more data. Therefore, the test becomes more powerful compared to the test that tends to make a final decision on $T_{2}$ earlier. This selection of bounds also corresponds to the smallest probability of rejecting the first hypothesis and not the second one (that is the probability of making an error when $\theta^{\left(2\right)}>0$) compared to the other combinations.

The combination that uses O'Brien and Fleming bounds for both treatment arms, the combination with Pocock bound for $T_{1}$ and O'Brien and Fleming bounds for $T_{2}$, and the combination of triangular and O'Brien and Fleming bounds result in similar power to reject both hypotheses but the first two combinations require lower maximum sample size compared to the latter—198 and 204 patients, respectively against 210 patients. At the same time, the three combinations differ in terms of ESS and in terms of probability of rejecting only the first hypothesis. Indeed, among these three combinations, the one with triangular bound for $T_{1}$ and O'Brien and Fleming bound for $T_{2}$ is the one with the smallest ESS and presents the smallest probability of rejecting only the first hypothesis.

The combination that uses O'Brien and Fleming bound for $T_{1}$ and triangular bound for $T_{2}$ is the one with smallest power and highest probability of rejecting only the first hypothesis. While the combination that uses triangular bounds for both treatment arms is the one with the smallest ESS (indeed triangular bounds are constructed to minimize the ESS^18^) for each configuration of θ compared to the other ones, even though it is one of the combinations with the highest maximum sample size—222 patients. Nevertheless, this combination has slightly smaller power of rejecting both hypotheses and slightly smaller probability of rejecting only the first hypothesis compared to the combination with Pocock bounds for $T_{1}$ and triangular bound for $T_{2}$.

Overall, the results suggest that there is a benefit, in terms of power and ESS, in using different bounds for each treatment arm. However, the final choice of the bounds will depend on the specific objectives of the trial. For example, in order to minimize the ESS it is recommended the use of triangular bounds for both treatment arms, whereas in order to maximize the power of rejecting all hypotheses it is recommended the use of triangular bounds for $T_{1}$ and O'Brien and Fleming bounds for $T_{2}$.

## 8 DISCUSSION

The aim of the current study was to explore MAMS designs that could select the most promising arm associated to the minimum treatment effect. An approach that takes the order relationship among the treatment effects (when no parametric dose‐response or duration‐response model is assumed) into account has been proposed. In the proposed approach we claim the effectiveness of the arm associated to the minimum treatment effect compared to the control only if we claim that the treatment associated to the maximum effect is efficacious. Through theoretical arguments and extensive numerical evaluation we show that the proposed design can provide noticeable advantages in power and/or expected sample sizes required in the trial compared to the alternatives.

The proposed design can be applied to a wide range of clinical trial settings where it could be assumed an order among the treatment effects. For example, in clinical trial designs applied to infectious diseases, such as TB and HBV, where it can be assumed that longer treatment durations correspond to a higher efficacy. In this case, the focus translates into the problem of selecting the shortest promising treatment duration. The proposed design can also be applied to clinical trial settings where nested combinations of treatments are tested against a common control arm.

The proposed design is closely linked to the hierarchical procedures described in the literature for example, by Glimm et al,^23^ Tamhane et al.^24^, ^25^ In particular, in the literature, the hierarchical procedure is applied for testing multiple endpoints in a specific order. In some applications, the interest is to test the secondary endpoint just if the primary endpoint has been rejected. Therefore, the endpoints could be tested on hierarchically order and various strategies can be adopted to test the hypotheses^23^ depending on the study objectives. It can be noted that when non‐binding futility boundaries ($l_{1}^{\left(1\right)}=l_{1}^{\left(2\right)}=-\infty$) are used in the 3‐arm 2‐stage ordered restricted design, the overall testing procedure^23^ coincides with the proposed method.

In this study, it has been assumed that the common variance is known. However, the effect of this assumption is not negligible especially with small sample sizes. In this case, a possible approach would be to transform the individual test statistics using the function $f(x)=\Phi^{-1}\{T_{d}(x)\}$, where *T* denotes the *t* distribution function with *d* degrees of freedom and Φ is the standard cumulative density function. More details of this approach are given in Jennison and Turnbull.^26^

Several avenues of future research present itself. First, focus has been given to superiority tests in this work. In certain diseases, such as in TB, non‐inferiority designs are the norm and hence further research on non‐inferiority hypothesis tests is of interest. Second, we assume that information time is the same for all treatments. When considering different durations of treatment, however, this information accumulates a different times and hence further work will consider optimal designs in this setting. Finally, we assume in this work that there is no uncertainty about the order of the treatment effects. Using a Bayesian framework, however, would naturally allow for uncertainty in that assumption to be considered.

## CONFLICT OF INTEREST

The authors declare no potential conflict of interests.

## AUTHOR CONTRIBUTIONS

All authors have directly participated in the planning and execution of the presented work.

## Supporting information

Data S1: Supplementary material

## Data Availability

Data sharing is not applicable to this article as no new data were created or analyzed in this study.
