# Supplementary material for: An order restricted multi‐arm multi‐stage clinical trial design
Source: Stat Med. 2022 Jan 19;41(9):1613–26. doi: 10.1002/sim.9314 (PMC7612618; doi:10.1002/sim.9314)
Supplement: Supplementary file 1 — Data S1: Supplementary material [file SIM-41-1613-s001.pdf]

Received: Added at production | Revised: Added at production | Accepted: Added at production
DOI: xxx/xxxx

ARTICLE TYPE

Supporting Information for ’An order restricted multi-arm multi-stage clinical trial design’

Alessandra Serra*[1] | Pavel Mozgunov[1] | Thomas Jaki[1,2]

[1]MRC Biostatistics Unit, University of Cambridge, United Kingdom
[2]Department of Mathematics and Statistics, Lancaster University, United Kingdom

Correspondence
*Corresponding author name, Email: alessandra.serra@mrc-bsu.cam.ac.uk

Present Address
This is sample for present address text this is sample for present address text

Summary

One family of designs that can noticeably improve efficiency in later stages of drug development are Multi-Arm Multi-Stage (MAMS) designs. They allow several arms to be studied concurrently and gain efficiency by dropping poorly performing treatment arms during the trial as well as by allowing to stop early for benefit. Conventional MAMS designs were developed for the setting, in which treatment arms are independent and hence can be inefficient when an order in the effects of the arms can be assumed (e.g. when considering different treatment durations or different doses). In this work, we extend the MAMS framework to incorporate the order of treatment effects when no parametric dose-response or duration-response model is assumed. The design can identify all promising treatments with high probability. We show that the design provides strong control of the family-wise error rate and illustrate the design in a study of symptomatic asthma. Via simulations we show that the inclusion of the ordering information leads to better decision-making compared to a fixed sample and a MAMS design. Specifically, in the considered settings, reductions in sample size of around 15% were achieved in comparison to a conventional MAMS design.

KEYWORDS:
Adaptive designs; Infectious diseases; Multi-arm Multi-stage; Order restriction.

1 | STRONG CONTROL OF THE FWER: K-ARM J-STAGE ORD

The 3-arm 2-stage design is a special case of the K-arm J-stage design. Thus, the proof of Theorem 1 logically follows from the proof of Theorem 2, which is given below.

Proof. We first consider the case where $\boldsymbol{\theta_0} = (\theta^{(1)}, \dots, \theta^{(K-1)})$, with $\theta^{(l)} \leq 0, l \in \{1, \dots, K-1\}$. Note that in this case the FWER is maximised when $\theta^{(1)} = 0$. Thus, under the worst case scenario $\boldsymbol{\theta_0} = (0, \theta^{(l)}, ..., \theta^{(K-1)})$, with $\theta^{(l)} \leq 0, l \in \{2, \dots, K-1\}$ it follows

$$\begin{aligned}&P(\text{rejecting at least one true } H_{0k},\ k \in \{1, ..., K-1\} \mid \boldsymbol{\theta_0}) = \\ &P(\text{reject } H_{01} \mid \boldsymbol{\theta_0}) = P(\text{reject } H_{01} \mid H_{01}) \leq \alpha.\end{aligned}$$

Let now consider $\boldsymbol{\theta_0} = (\theta^{(1)}, ..., \theta^{(n)}, \theta^{(n+1)}, ..., \theta^{(K-1)})$, with $\theta^{(s)} > 0, s \in \{1, ..., n\}$ and $\theta^{(l)} \leq 0, l \in \{n+1, ..., K-1\}$. Note that the FWER under $\boldsymbol{\theta_0}$, with $\theta^{(s)} > 0, s \in \{1, ..., n\}$ and $\theta^{(l)} \leq 0, l \in \{n+1, ..., K-1\}$ is maximized when $n + 1 = 2$ and

$\theta^{(l)} = 0, l \in \{2, ..., K-1\}$. Therefore, lets consider the worst case scenario $\boldsymbol{\theta_0} = (\theta^{(1)}, 0, ..., 0)$, with $\theta^{(1)} > 0$ and denote with $\boldsymbol{\Theta_L}$ a vector of zeros of length $K-2$. Then

$$P(\text{rejecting at least one true } H_{0k},\ k \in \{1, ..., K-1\} \mid \boldsymbol{\theta_0} = (\theta^{(1)}, 0, ..., 0)) =$$
$$P(\text{reject } H_{02} \mid \boldsymbol{\theta_0}) = P(\text{reject } H_{02} \mid \text{reject } H_{01}, \boldsymbol{\theta_0}) \times P(\text{reject } H_{01} \mid \boldsymbol{\theta_0}) \leq$$
$$P(\text{reject } H_{02} \mid \text{reject } H_{01}, \overline{\boldsymbol{\theta_0}} = (\infty, 0, \ldots, 0)).$$

If we denote the vector of Z-statistics under H_0 with

$$\mathbf{Z_{1,s}} \sim N_{J\times(K-1)}(\boldsymbol{\mu_{1,s}}, \boldsymbol{\Sigma_{1,s}}), s \in \{1, ..., K-1\}$$

and the vector of Z-statistics under $\boldsymbol{\Theta_L}$ with

$$\mathbf{Z_{2,l}} \sim N_{J\times(K-2)}(\boldsymbol{\mu_{2,l}}, \boldsymbol{\Sigma_{2,l}}), l \in \{2, ..., K-1\}.$$

Then given that $u_j^{(k)} = u_j$, $l_j^{(k)} = l_j$ and $r_j^{(k)} = r_j$, for all $k \in \{1, ..., K-1\}$, it follows that

$$\boldsymbol{\Sigma_{1,\bar{s}}} = \boldsymbol{\Sigma_{2,l}},$$

with $\bar{s} \in \{1, ..., K-2\}$. Therefore

$$P(\text{reject } H_{02} \mid \text{reject } H_{01}, \overline{\boldsymbol{\theta_0}} = (\infty, 0, \ldots, 0)) =$$
$$P\left(Z_1^{(1)} \geq u_1 \mid \theta^{(1)} = 0\right) + \sum_{j=2}^{J}\sum_{m=2}^{K-1} P\left(Z_j^{(1)} \geq u_j \mid M_j = m, \boldsymbol{\Theta_L}\right) \times P\left(M_j = m\right) <$$
$$P\left(Z_1^{(1)} \geq u_1 \mid \theta^{(1)} = 0\right) + \sum_{j=2}^{J}\sum_{m=2}^{K} P\left(Z_j^{(1)} \geq u_j \mid M_j = m, H_0\right) \times P\left(M_j = m\right) =$$
$$P(\text{rejecting at least one true } H_{0k},\ k \in \{1, ..., K-1\} \mid H_0) \leq \alpha$$

completing the proof. □

2 | DECISION RULES FOR THE 3-ARM 2-STAGE DESIGN

Other decision rules at the interim analyses could be considered by the proposed design. As in Section 3 of the main body of the text, we consider the 3-arm 2-stage example. We consider two different alternatives described in Table 1 and Table 2.

The first alternative of decision rule (Table 1) differs from the one described in Section 3 in the main paper in the cells coloured in red. For the described combination of the decision rules, the equation for the FWER is:

$$P\left(Z_1^{(1)} \geq u_1^{(1)} \mid H_0\right) + P\left(Z_2^{(1)} \geq u_2^{(1)}, l_1^{(1)} < Z_1^{(1)} < u_1^{(1)} \mid H_0\right) \tag{1}$$

The power equation to reject both hypotheses is:

$$\begin{aligned} &P\left(Z_1^{(1)} \geq u_1^{(1)}, Z_1^{(2)} \geq u_1^{(2)} \mid \boldsymbol{\theta}\right) + \\ &P\left(Z_2^{(2)} \geq u_2^{(2)}, Z_1^{(1)} \geq u_1^{(1)}, l_1^{(2)} < Z_1^{(2)} < u_1^{(2)} \mid \boldsymbol{\theta}\right) + \\ &P\left(Z_2^{(1)} \geq u_2^{(1)}, Z_2^{(2)} \geq u_2^{(2)}, l_1^{(1)} < Z_1^{(1)} < u_1^{(1)}, Z_1^{(2)} \geq l_1^{(2)} \mid \boldsymbol{\theta}\right) \end{aligned} \tag{2}$$

The second alternative (Table 2) differs from the original described in the main paper in the red cells. For the described combination of the decision rules, the equation for the FWER is:

$$\begin{aligned} &P\left(Z_1^{(1)} \geq u_1^{(1)} \mid H_0\right) + P\left(Z_2^{(1)} \geq u_2^{(1)}, l_1^{(1)} < Z_1^{(1)} < u_1^{(1)} \mid H_0\right) + \\ &P\left(Z_2^{(1)} \geq u_2^{(1)}, Z_1^{(1)} \leq l_1^{(1)}, Z_1^{(2)} \geq l_1^{(2)} \mid H_0\right) \end{aligned} \tag{3}$$

The power equation to reject both hypotheses is:

$$P\left(Z_1^{(1)} \geq u_1^{(1)}, Z_1^{(2)} \geq u_1^{(2)} \mid \theta\right) + P\left(Z_2^{(2)} \geq u_2^{(2)}, Z_1^{(1)} \geq u_1^{(1)}, l_1^{(2)} < Z_1^{(2)} < u_1^{(2)} \mid \theta\right) + P\left(Z_2^{(1)} \geq u_2^{(1)}, Z_2^{(2)} \geq u_2^{(2)}, Z_1^{(1)} < u_1^{(1)}, Z_1^{(2)} \geq l_1^{(2)} \mid \theta\right) \quad (4)$$

We compare the three decision rules using triangular[1] boundaries and considering the same bounds for both treatments $u_1^{(1)} = u_1^{(2)} = u_1$ and $l_1^{(1)} = l_1^{(2)} = l_1$. The design is powered at 80% to reject both hypotheses. The Pocock[2] and O'Brien & Fleming[3] boundaries for both treatments were considered as well but the difference in the bounds and the sample size were negligible.

The difference in power among the three different combinations of decision rules are reported in Figure 1 with the following notation:

- decision rule 1 (decrule1): stop the trial when $Z_1^{(1)} \leq l_1, Z_1^{(2)} \geq u_1$ and $Z_1^{(1)} \leq l_1, l_1 < Z_1^{(2)} < u_1$;
- decision rule 2 (decrule2): continue the trial when $Z_1^{(1)} \leq l_1, Z_1^{(2)} \geq u_1$ and $Z_1^{(1)} \leq l_1, l_1 < Z_1^{(2)} < u_1$;
- decision rule 3 (decrule3): continue the trial when $Z_1^{(1)} \leq l_1, Z_1^{(2)} \geq u_1$ and stop the trial when $Z_1^{(1)} \leq l_1, l_1 < Z_1^{(2)} < u_1$.

No major differences are observed between the three different decision rules in Figure 1, because the probability of rejecting both hypotheses is quite similar for all the three decision rules. Furthermore, the differences in the ESS are negligible (the three designs differ in only one patient per arm per stage). It follows that, also when the triangular bounds are used at the interim analyses, the different decision rules present minimal differences on the power and the ESS. Thus, one could decide which rules to use depending on the clinical context.

3 | POWER COMPARISON ORD AND FSD

Lets consider a 3-arm 2-stage ORD. Let $u_1^{(1)} = u_1^{(2)} = u_2^{(1)} = u_2^{(2)} = u_1$, $l_1^{(1)} = l_1^{(2)} = l_1 = -u_1$ be the critical values such that Equation (1) holds under the global null hypothesis. Assume that the interim analysis is done after half of the total sample size has been observed and consider an equal allocation ratio to all arms. Let u be the critical bound for the fixed balanced sample design (FSD) and n the sample size per arm per stage and assume $\sigma = 1$.

Lemma 1 states that, under these assumptions, it follows that $u_1 < u$ so that the 3-arm 2-stage ORD is always more powerful that the FSD with the same total sample size.

Lemma 1. Consider a 3-arm 2-stage ORD and denote the global null hypothesis by $H_0 : \theta^{(1)} = \theta^{(2)} = 0$. Let $u_1^{(1)} = u_1^{(2)} = u_2^{(1)} = u_2^{(2)} = l_2^{(1)} = l_2^{(2)} = u_1$, $l_1^{(1)} = l_1^{(2)} = l_1 = -u_1$ be the critical values such that Equation (1) holds under the global null hypothesis. Let u the critical bound for the FSD. Under these assumptions $u_1 < u$.

Proof. Lemma 1 is proven by contradiction. Assume that $u_1 \geq u$.

For the ORD, the critical values are found to satisfy the following equality

$$P\left(Z_1^{(1)} \geq u_1\right) + P\left(Z_2^{(1)} \geq u_1, l_1 < Z_1^{(1)} < u_1\right) + P\left(Z_2^{(1)} \geq u_1, Z_1^{(1)} \leq l_1, Z_1^{(2)} \geq u_1\right) = \alpha \quad (5)$$

under H_0. For the FSD the critical bound u is found in order to satisfy

$$P\left(Z_1^{(1)} \geq u\right) + P\left(Z_1^{(2)} \geq u\right) - P\left(Z_1^{(1)} \geq u, Z_1^{(2)} \geq u\right) = \alpha \quad (6)$$

under H_0. Therefore,

$$P\left(Z_1^{(1)} \geq u_1\right) + P\left(Z_2^{(1)} \geq u_1, l_1 < Z_1^{(1)} < u_1\right) + P\left(Z_2^{(1)} \geq u_1, Z_1^{(1)} \leq l_1, Z_1^{(2)} \geq u_1\right) - P\left(Z_1^{(1)} \geq u\right) - P\left(Z_1^{(2)} \geq u\right) + P\left(Z_1^{(1)} \geq u, Z_1^{(2)} \geq u\right) = 0 \quad (7)$$

and if $u_1 \geq u$ then

$$P\left(Z_1^{(1)} < u, Z_1^{(2)} < u\right) \leq P\left(Z_1^{(1)} < u_1, Z_1^{(2)} < u_1\right).$$

Therefore from Equation (7), it follows that

$$P\left(Z_1^{(1)} \geq u_1\right) + P\left(Z_2^{(1)} \geq u_1, l_1 < Z_1^{(1)} < u_1\right) + P\left(Z_2^{(1)} \geq u_1, Z_1^{(1)} \leq l_1, Z_1^{(2)} \geq u_1\right) +$$
$$P\left(Z_1^{(1)} < u, Z_1^{(2)} < u\right) - 1 \leq$$
$$P\left(Z_1^{(1)} \geq u_1\right) + P\left(Z_2^{(1)} \geq u_1, l_1 < Z_1^{(1)} < u_1\right) + P\left(Z_2^{(1)} \geq u_1, Z_1^{(1)} \leq l_1, Z_1^{(2)} \geq u_1\right) +$$
$$P\left(Z_1^{(1)} < u_1, Z_1^{(2)} < u_1\right) - 1 =$$
$$1 - P\left(Z_1^{(1)} \leq l_1, Z_1^{(2)} < u_1\right) - P\left(Z_2^{(1)} < u_1, Z_1^{(1)} < u_1, Z_1^{(2)} \geq u_1\right) -$$
$$P\left(Z_2^{(1)} < u_1, l_1 < Z_1^{(1)} < u_1, l_1 < Z_1^{(2)} < u_1\right) - P\left(Z_2^{(1)} < u_1, l_1 < Z_1^{(1)} < u_1, Z_1^{(2)} \leq l_1\right) +$$
$$P\left(Z_1^{(1)} < u_1, Z_1^{(2)} < u_1\right) - 1 \tag{8}$$

Using Slepian theorem[4], one can show that Equation (8) is equal to

$$-P\left(Z_2^{(1)} < u_1, Z_1^{(1)} < u_1\right) + P\left(Z_1^{(1)} < u_1, Z_1^{(2)} < u_1\right) - P\left(Z_2^{(1)} \geq u_1, Z_1^{(1)} < l_1, Z_1^{(2)} < u_1\right) < 0$$

which is a contradiction and therefore the Lemma is proven. □

Furthermore, under the assumptions of Lemma 1, numerical evaluations show that $P(\text{rejecting } H_{01} \text{ and } H_{02}|_{ORD}) - P(\text{rejecting } H_{01} \text{ and } H_{02}|_{FSD}) > 0$, when $\theta^{(1)}, \theta^{(2)} \in (0, 2), \alpha \in \{0.05, 0.1\}$ and when the sample size per arm for the FSD consists of 10 or 50 patients.

Let n_1 be the number of patients per arm at the first stage and n_2 at the second stage. From Equation (2), the $P(\text{rejecting } H_{01} \text{ and } H_{02}|\boldsymbol{\theta})$ for a 3-arm 2-stage ORD design can be written as:

$$P\left(N_1^{(1)} \geq u_1 - \frac{\theta^{(1)}\sqrt{2n_1}}{2\sigma}, N_1^{(2)} \geq u_1 - \frac{\theta^{(2)}\sqrt{2n_1}}{2\sigma}\right) +$$
$$P\left(N_2^{(1)} \geq u_1 - \frac{\theta^{(1)}\sqrt{2n_2}}{2\sigma}, N_2^{(2)} \geq u_1 - \frac{\theta^{(2)}\sqrt{2n_2}}{2\sigma}, N_1^{(1)} < u_1 - \frac{\theta^{(1)}\sqrt{2n_1}}{2\sigma}, N_1^{(2)} \geq u_1 - \frac{\theta^{(2)}\sqrt{2n_1}}{2\sigma}\right) +$$
$$P\left(N_2^{(2)} \geq u_1 - \frac{\theta^{(2)}\sqrt{2n_2}}{2\sigma}, N_1^{(1)} \geq u_1 - \frac{\theta^{(1)}\sqrt{2n_1}}{2\sigma}, l_1 - \frac{\theta^{(2)}\sqrt{2n_1}}{2\sigma} < N_1^{(2)} < u_1 - \frac{\theta^{(2)}\sqrt{2n_1}}{2\sigma}\right) +$$
$$P\left(N_2^{(1)} \geq u_1 - \frac{\theta^{(1)}\sqrt{2n_2}}{2\sigma}, N_2^{(2)} \geq u_1 - \frac{\theta^{(2)}\sqrt{2n_2}}{2\sigma}, l_1 - \frac{\theta^{(1)}\sqrt{2n_1}}{2\sigma} < N_1^{(1)} < u_1 - \frac{\theta^{(1)}\sqrt{2n_1}}{2\sigma},\right.$$
$$\left. l_1 - \frac{\theta^{(2)}\sqrt{2n_1}}{2\sigma} < N_1^{(2)} < u_1 - \frac{\theta^{(2)}\sqrt{2n_1}}{2\sigma}\right)$$

where

$$\begin{pmatrix} N_1^{(1)} \\ N_1^{(2)} \\ N_2^{(1)} \\ N_2^{(2)} \end{pmatrix} \sim N\left[\begin{pmatrix} 0 \\ 0 \\ 0 \\ 0 \end{pmatrix}, \begin{pmatrix} 1 & \frac{1}{2} & \sqrt{\frac{n_1}{n_2}} & \frac{1}{2}\sqrt{\frac{n_1}{n_2}} \\ \frac{1}{2} & 1 & \frac{1}{2}\sqrt{\frac{n_1}{n_2}} & \sqrt{\frac{n_1}{n_2}} \\ \sqrt{\frac{n_1}{n_2}} & \frac{1}{2}\sqrt{\frac{n_1}{n_2}} & 1 & \frac{1}{2} \\ \frac{1}{2}\sqrt{\frac{n_1}{n_2}} & \sqrt{\frac{n_1}{n_2}} & \frac{1}{2} & 1 \end{pmatrix}\right]$$

For the FSD with n patients per arm it holds

$$P(\text{rejecting } H_{01} \text{ and } H_{02}|\boldsymbol{\theta}) = P\left(\overline{N}^{(1)} \geq u - \frac{\theta^{(1)}\sqrt{2n}}{2\sigma}, \overline{N}^{(2)} \geq u - \frac{\theta^{(2)}\sqrt{2n}}{2\sigma}\right)$$

where

$$\begin{pmatrix} \overline{N}^{(1)} \\ \overline{N}^{(2)} \end{pmatrix} \sim N\left[\begin{pmatrix} 0 \\ 0 \end{pmatrix}, \begin{pmatrix} 1 & \frac{1}{2} \\ \frac{1}{2} & 1 \end{pmatrix}\right]$$

Let $\sigma = 1$ and fix the maximum sample size for both designs. Therefore, if n is the sample size per arm for the FSD, then $n_1 = \frac{n}{2}$ and if $n_1 = 2n_2$ then $n_2 = n$. Given that

$$\begin{pmatrix} \overline{N}^{(1)} \\ \overline{N}^{(2)} \end{pmatrix} \sim \begin{pmatrix} N_2^{(1)} \\ N_2^{(2)} \end{pmatrix}$$

it follows

$$\begin{aligned}
&P(\text{rejecting } H_{01} \text{ and } H_{02}|_{ORD}) - P(\text{rejecting } H_{01} \text{ and } H_{02}|_{FSD}) = \\
&P\left(N_1^{(1)} \geq u_1 - \frac{\theta^{(1)}\sqrt{n}}{2}, N_1^{(2)} \geq u_1 - \frac{\theta^{(2)}\sqrt{n}}{2}\right) + \\
&P\left(N_2^{(1)} \geq u_1 - \frac{\theta^{(1)}\sqrt{2n}}{2}, N_2^{(2)} \geq u_1 - \frac{\theta^{(2)}\sqrt{2n}}{2}, N_1^{(1)} < u_1 - \frac{\theta^{(1)}\sqrt{n}}{2}, N_1^{(2)} \geq u_1 - \frac{\theta^{(2)}\sqrt{n}}{2}\right) + \\
&P\left(N_2^{(2)} \geq u_1 - \frac{\theta^{(2)}\sqrt{2n}}{2}, N_1^{(1)} \geq u_1 - \frac{\theta^{(1)}\sqrt{n}}{2}, l_1 - \frac{\theta^{(2)}\sqrt{n}}{2} < N_1^{(2)} < u_1 - \frac{\theta^{(2)}\sqrt{n}}{2}\right) + \\
&P\left(N_2^{(1)} \geq u_1 - \frac{\theta^{(1)}\sqrt{2n}}{2}, N_2^{(2)} \geq u_1 - \frac{\theta^{(2)}\sqrt{2n}}{2}, l_1 - \frac{\theta^{(1)}\sqrt{n}}{2} < N_1^{(1)} < u_1 - \frac{\theta^{(1)}\sqrt{n}}{2},\right. \\
&\left. l_1 - \frac{\theta^{(2)}\sqrt{n}}{2} < N_1^{(2)} < u_1 - \frac{\theta^{(2)}\sqrt{n}}{2}\right) - P\left(N_2^{(1)} \geq u - \frac{\theta^{(1)}\sqrt{2n}}{2}, N_2^{(2)} \geq u - \frac{\theta^{(2)}\sqrt{2n}}{2}\right)
\end{aligned}$$

Using Lemma 1, it holds

$$\begin{aligned}
&P(\text{rejecting } H_{01} \text{ and } H_{02}|_{ORD}) - P(\text{rejecting } H_{01} \text{ and } H_{02}|_{FSD}) > \\
&P\left(N_1^{(1)} \geq u_1 - \frac{\theta^{(1)}\sqrt{n}}{2}, N_1^{(2)} \geq u_1 - \frac{\theta^{(2)}\sqrt{n}}{2}\right) + \\
&P\left(N_2^{(1)} \geq u_1 - \frac{\theta^{(1)}\sqrt{2n}}{2}, N_2^{(2)} \geq u_1 - \frac{\theta^{(2)}\sqrt{2n}}{2}, N_1^{(1)} < u_1 - \frac{\theta^{(1)}\sqrt{n}}{2}, N_1^{(2)} \geq u_1 - \frac{\theta^{(2)}\sqrt{n}}{2}\right) + \\
&P\left(N_2^{(2)} \geq u_1 - \frac{\theta^{(2)}\sqrt{2n}}{2}, N_1^{(1)} \geq u_1 - \frac{\theta^{(1)}\sqrt{n}}{2}, l_1 - \frac{\theta^{(2)}\sqrt{n}}{2} < N_1^{(2)} < u_1 - \frac{\theta^{(2)}\sqrt{n}}{2}\right) + \\
&P\left(N_2^{(1)} \geq u_1 - \frac{\theta^{(1)}\sqrt{2n}}{2}, N_2^{(2)} \geq u_1 - \frac{\theta^{(2)}\sqrt{2n}}{2}, l_1 - \frac{\theta^{(1)}\sqrt{n}}{2} < N_1^{(1)} < u_1 - \frac{\theta^{(1)}\sqrt{n}}{2},\right. \\
&\left. l_1 - \frac{\theta^{(2)}\sqrt{n}}{2} < N_1^{(2)} < u_1 - \frac{\theta^{(2)}\sqrt{n}}{2}\right) - P\left(N_2^{(1)} \geq u_1 - \frac{\theta^{(1)}\sqrt{2n}}{2}, N_2^{(2)} \geq u_1 - \frac{\theta^{(2)}\sqrt{2n}}{2}\right)
\end{aligned}$$

Through some further analytical passages it can be shown that

$$\begin{aligned}
&P(\text{rejecting } H_{01} \text{ and } H_{02}|_{ORD}) - P(\text{rejecting } H_{01} \text{ and } H_{02}|_{FSD}) > \\
&P\left(N_2^{(2)} < u_1 - \frac{\theta^{(2)}\sqrt{2n}}{2}, N_1^{(1)} \geq u_1 - \frac{\theta^{(1)}\sqrt{n}}{2}, N_1^{(2)} \geq u_1 - \frac{\theta^{(2)}\sqrt{n}}{2}\right) + \\
&P\left(N_2^{(1)} < u_1 - \frac{\theta^{(1)}\sqrt{2n}}{2}, N_2^{(2)} \geq u_1 - \frac{\theta^{(2)}\sqrt{2n}}{2}, N_1^{(1)} > u_1 - \frac{\theta^{(1)}\sqrt{n}}{2}, N_1^{(2)} > l_1 - \frac{\theta^{(2)}\sqrt{n}}{2}\right) + \\
&P\left(N_2^{(1)} \geq u_1 - \frac{\theta^{(1)}\sqrt{2n}}{2}, N_2^{(2)} \geq u_1 - \frac{\theta^{(2)}\sqrt{2n}}{2}, N_1^{(1)} < l_1 - \frac{\theta^{(1)}\sqrt{n}}{2}, N_1^{(2)} > u_1 - \frac{\theta^{(2)}\sqrt{n}}{2}\right) + \\
&- P\left(N_2^{(1)} \geq u_1 - \frac{\theta^{(1)}\sqrt{2n}}{2}, N_2^{(2)} \geq u_1 - \frac{\theta^{(2)}\sqrt{2n}}{2}, N_1^{(1)} < l_1 - \frac{\theta^{(1)}\sqrt{n}}{2}\right) +
\end{aligned}$$

$$- P\left(N_2^{(1)} \geq u_1 - \frac{\theta^{(1)}\sqrt{2n}}{2}, N_2^{(2)} \geq u_1 - \frac{\theta^{(2)}\sqrt{2n}}{2}, N_1^{(1)} > l_1 - \frac{\theta^{(1)}\sqrt{n}}{2}, N_1^{(2)} < l_1 - \frac{\theta^{(2)}\sqrt{n}}{2} \right) \tag{9}$$

Given the analytical complexity of Equation (9), we evaluate it numerically using R, choosing $\alpha = 0.05$ and $u_1 = 1.876$, $\alpha = 0.1$ and $u_1 = 1.527$. Moreover, we consider $n = 10, 50$ and $\theta^{(1)} \in (0, 2), \theta^{(2)} \in (0, 2)$. Figure 2 shows that the difference in power to reject both hypotheses between the ORD and the FSD is always greater than zero for the chosen values of $u_1, \boldsymbol{\theta}$ and for the considered sample sizes.

4 | K-ARM 2-STAGE DESIGN

Consider a clinical trial with $K - 1, K \geq 4$ active treatment arms, $T_1, ..., T_{K-1}$, against a control treatment and 2 stages. The FWER can be found recursively as

$$\begin{aligned} &P(\text{rejecting at least one true } H_{0k},\ k \in \{1, ..., K-1\} \mid H_0) = \\ &P(\text{rejecting at least one true } H_{0k} \text{ in a } (K-1)\text{-arm 2-stage design} \mid H_0) + \\ &P\left(Z_2^{(1)} \geq u_2^{(1)}, Z_1^{(1)} \leq l_1^{(1)}, \bigcap_{k=2}^{K-2} Z_1^{(k)} \leq u_1^{(k)}, Z_1^{(K-1)} \geq u_1^{(K-1)} \mid H_0 \right). \end{aligned} \tag{10}$$

5 | DIFFERENT BOUNDS FOR EACH TREATMENT ARM

Figure 3 shows the probability of rejecting both hypotheses under $\boldsymbol{\theta} = (0.5, \theta^{(2)})$ and $\theta^{(2)} \in \{0, 0.1, 0.2, 0.3, 0.4, 0.5\}$ for the 3-arm 2-stage ORD design when it is powered at 80% to reject both hypotheses under $\boldsymbol{\theta} = (0.5, 0.5)$. ORD uses different combination of bounds which control the type I error under $\boldsymbol{\theta} = (\infty, 0)$. Results are provided using 10^6 replications.

6 | CASE STUDY: NUMERICAL RESULTS

The results of the simulations that revisit the NCT01257230 trial using the ORD when 1 interim analysis is planned after observing half of the total population are provided in Table 3.

7 | COMPARISON OF THE CRITICAL VALUES BETWEEN A 3-ARM 2-STAGE ORD AND A STANDARD MAMS

In Figure 4 it can be seen how the values of different shape of critical bounds differ between the 3-arm 2-stage ORD and the standard MAMS design which select all promising arms to proceed to the next stage. The bounds for the ORD are found in order to control Equation (1) under H_0 at level $\alpha = 0.05$, when the same bound $(u_j, l_j), j \in \{1, 2\}$, are used for each treatment arm, whereas bounds for the MAMS design are found using the R[5] package proposed by Jaki et al.[6] It is worth noting that overall, the critical bounds for the ORD are smaller in each stage compared to the standard MAMS.

ACKNOWLEDGMENTS

This report is independent research supported by the National Institute for Health Research (NIHR Advanced Fellowship, Dr Pavel Mozgunov, NIHR300576; and Prof Jaki's Senior Research Fellowship, NIHR-SRF-2015-08-001) and by the NIHR Cambridge Biomedical Research Centre (BRC-1215-20014). The views expressed in this publication are those of the authors and not necessarily those of the NHS, the National Institute for Health Re- search or the Department of Health and Social Care (DHSC). T Jaki also received funding from UK Medical Research Council (MC_UU_00002/14).

Author contributions

All authors have directly participated in the planning and execution of the presented work.

Financial disclosure

None reported.

Conflict of interest

The authors declare no potential conflict of interests.

References

1. Whitehead J. *The design and analysis of sequential clinical trials*. John Wiley & Sons . 1997.
2. Pocock SJ. Group Sequential Methods in the Design and Analysis of Clinical Trials. *Biometrika* 1977; 64: 191. doi: 10.2307/2335684
3. O'Brien PC, Fleming TR. A Multiple Testing Procedure for Clinical Trials. *Biometrics* 1979; 35: 549. doi: 10.2307/2530245
4. Slepian D. The One-Sided Barrier Problem for Gaussian Noise. *Bell System Technical Journal* 1962; 41: 463-501. doi: 10.1002/j.1538-7305.1962.tb02419.x
5. R Core Team . *R: A Language and Environment for Statistical Computing*. R Foundation for Statistical Computing; Vienna, Austria: 2019.
6. Jaki T, Pallmann P, Magirr D. The R package MAMS for designing multi-arm multi-stage clinical trials. *Journal of Statistical Software* 2019; 88: 1-25. doi: 10.18637/jss.v088.i04

How to cite this article:

TABLE 1 Alternative 1 for the combination of the decision rules for a 3-arm 2-stage design with $\theta^{(1)} \geq \theta^{(2)}$. Cells coloured in red correspond to different decision rules compared to the ones described in Section 3 in the main paper.

	$Z_1^{(1)} \geq u_1^{(1)}$	$l_1^{(1)} < Z_1^{(1)} < u_1^{(1)}$	$Z_1^{(1)} \leq l_1^{(1)}$
$Z_1^{(2)} \geq u_1^{(2)}$	stop: select T_1, T_2	proceed with T_1, T_2	drop both arms
$l_1^{(2)} < Z_1^{(2)} < u_1^{(2)}$	proceed with T_2	proceed with T_1, T_2	drop both arms
$Z_1^{(2)} \leq l_1^{(2)}$	stop: select T_1	proceed with T_1	drop both arms

TABLE 2 Alternative 2 for the combination of the decision rules for a 3-arm 2-stage design with $\theta^{(1)} \geq \theta^{(2)}$. Cells coloured in red correspond to different decision rules compared to the ones described in Section 3 in the main paper.

	$Z_1^{(1)} \geq u_1^{(1)}$	$l_1^{(1)} < Z_1^{(1)} < u_1^{(1)}$	$Z_1^{(1)} \leq l_1^{(1)}$
$Z_1^{(2)} \geq u_1^{(2)}$	stop: select T_1, T_2	proceed with T_1, T_2	proceed with T_1, T_2
$l_1^{(2)} < Z_1^{(2)} < u_1^{(2)}$	proceed with T_2	proceed with T_1, T_2	proceed with T_1, T_2
$Z_1^{(2)} \leq l_1^{(2)}$	stop: select T_1	proceed with T_1	drop both arms

TABLE 3 Results of the simulations that revisit the NCT01257230 trial using the ORD when 1 interim analysis is planned after observing half of the total population. Constant (POC) and O'Brien & Fleming (OBF) bounds are used. Values of interest are in bold. Proportions refer to 10^6 replications. ESS: Expected Sample Size. Max. SS: Maximum sample size.

Design powered to reject all hypotheses							
$\theta^{(1)}$	$\theta^{(2)}$	Bounds	Max. SS	Reject all	Reject H_{01} not H_{02}	Reject at least one H_{0k}	ESS
0	0	POC	528	0.004	0.021	0.025	521.32
		OBF	480	0.004	0.021	0.025	478.97
120	0	POC	528	0.024	0.859	0.884	474.52
		OBF	480	0.025	0.857	0.882	456.52
120	120	POC	528	**0.805**	0.079	0.884	407.87
		OBF	480	**0.805**	0.077	0.882	433.68
Design powered to reject at least one hypothesis							
$\theta^{(1)}$	$\theta^{(2)}$	Bounds	Max. SS	Reject all	Reject H_{01} not H_{02}	Reject at least one H_{0k}	ESS
0	0	POC	426	0.004	0.021	0.025	420.68
		OBF	384	0.004	0.020	0.025	383.15
120	0	POC	426	0.024	0.783	0.807	389.54
		OBF	384	0.024	0.778	0.803	370.02
120	120	POC	426	0.692	0.114	**0.806**	349.59
		OBF	384	0.691	0.113	**0.804**	358.27

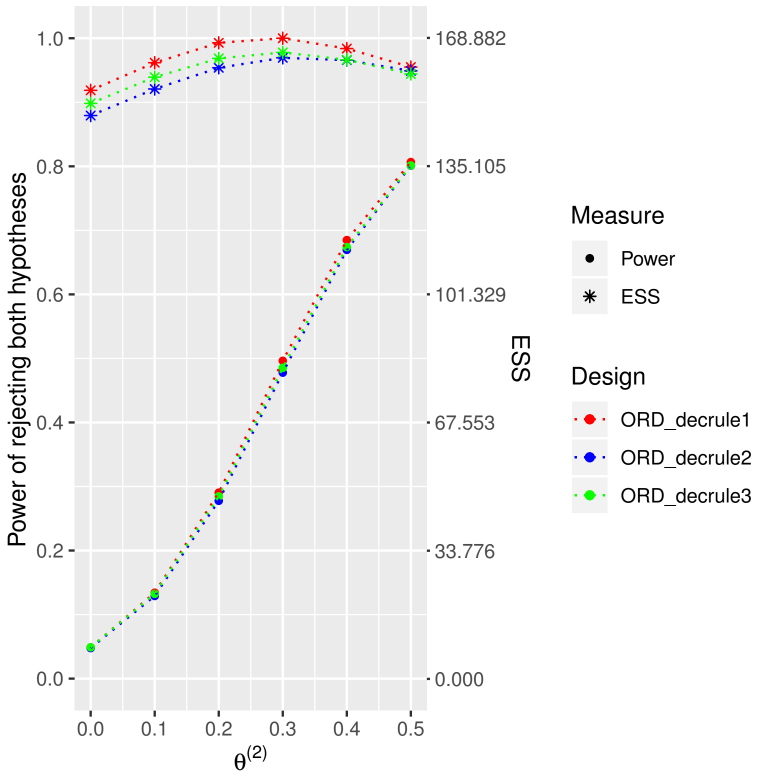

FIGURE 1 Probability of rejecting both hypotheses under $\boldsymbol{\theta} = (0.5, \theta^{(2)})$ and $\theta^{(2)} \in \{0, 0.1, 0.2, 0.3, 0.4, 0.5\}$ for the 3-arm 2-stage ORD design when it is powered at 80% to reject both hypotheses under $\boldsymbol{\theta} = (0.5, 0.5)$. ORD uses triangular bounds. Results are provided using 10^6 replications.

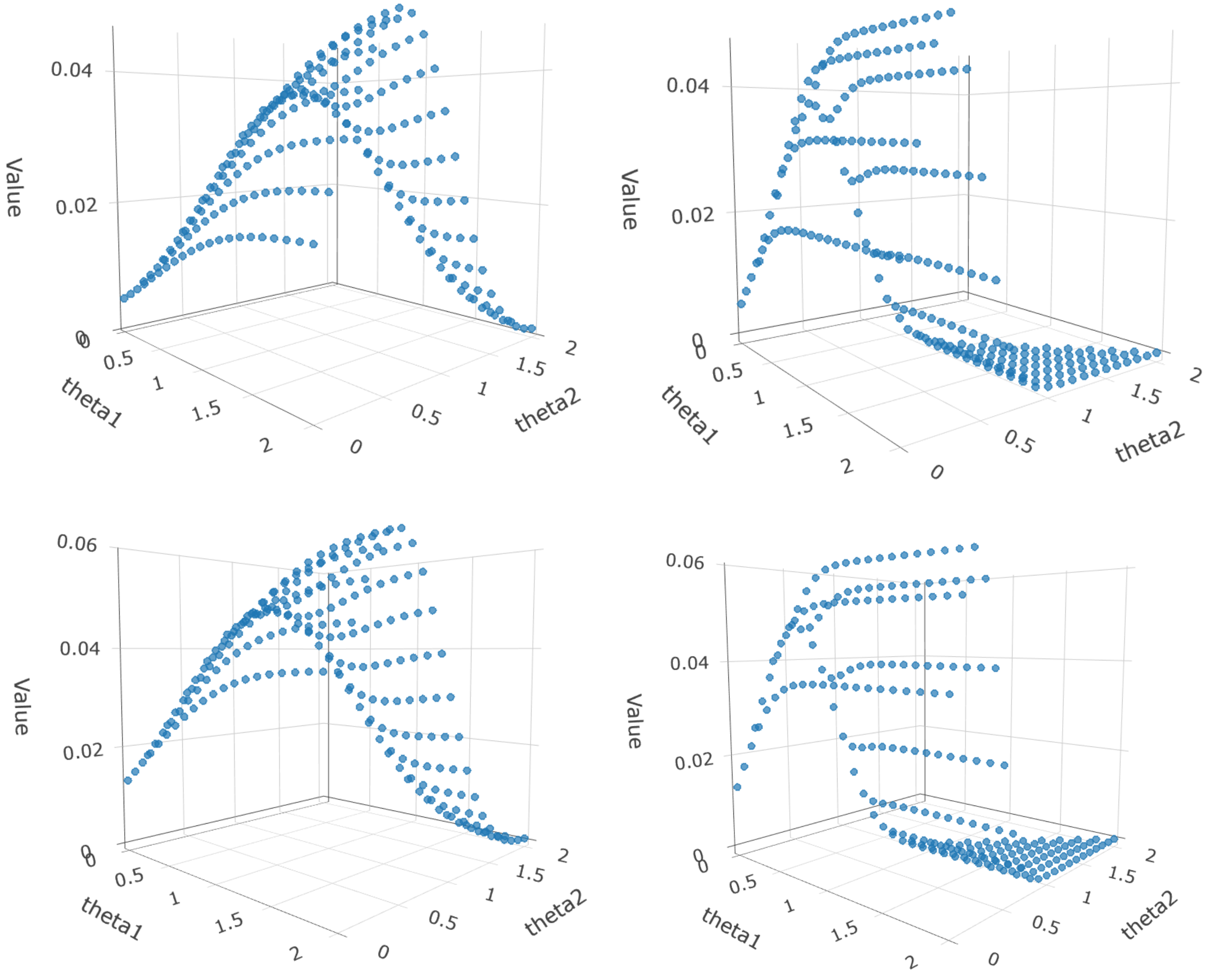

FIGURE 2 Numerical values of Equation (9) using different values of $\theta^{(1)}$ (theta1) and $\theta^{(2)}$ (theta2) when $u_1 = 1.876$ (figures in the first row), $u_1 = 1.527$ (figures in the second row) and $n = 10$ (column on the left), $n = 50$ (column on the right). Computations are obtained using R[5].

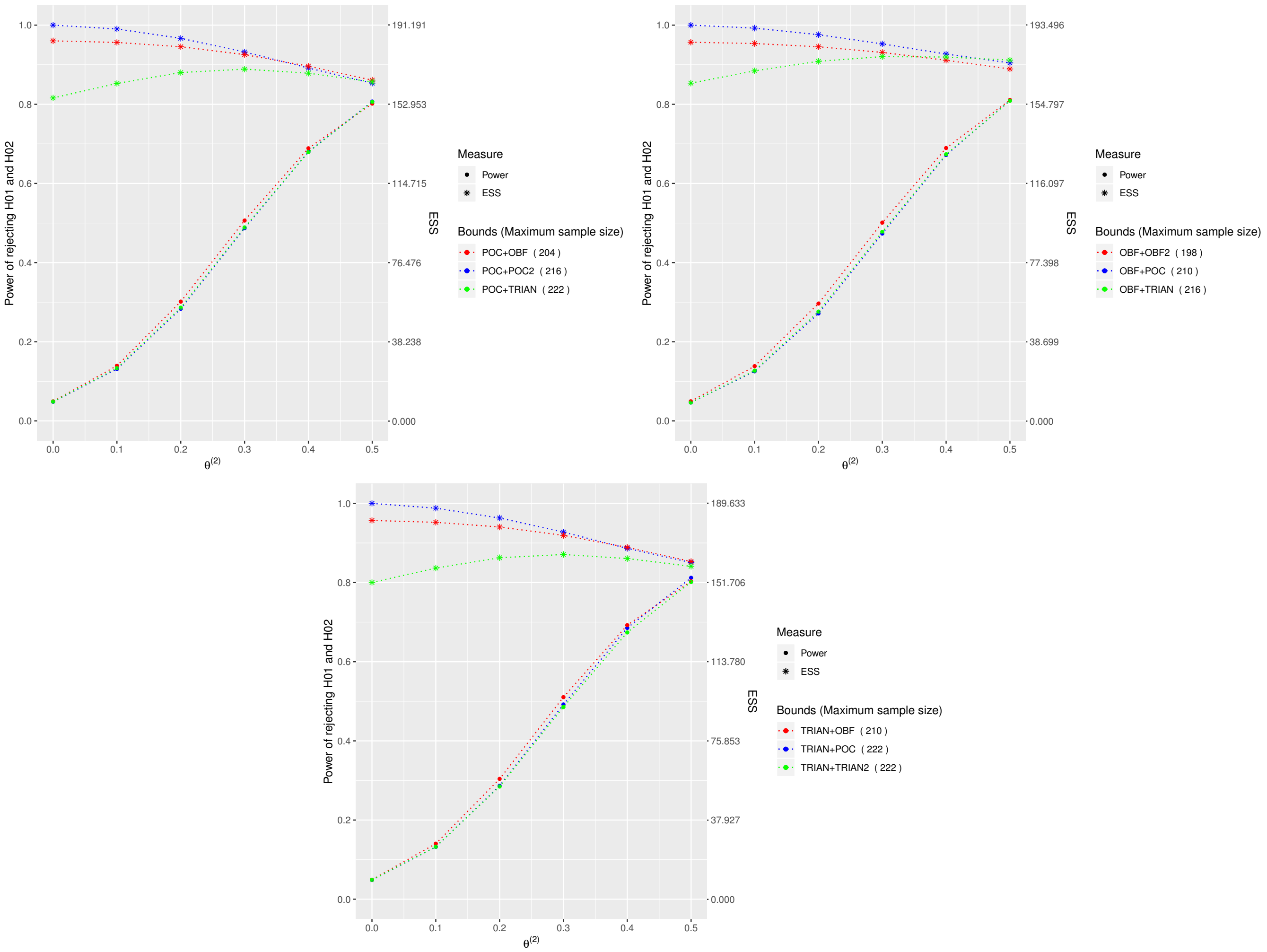

FIGURE 3 Probability of rejecting both hypotheses under $\boldsymbol{\theta} = (0.5, \theta^{(2)})$ and $\theta^{(2)} \in \{0, 0.1, 0.2, 0.3, 0.4, 0.5\}$ for the 3-arm 2-stage ORD design when it is powered at 80% to reject both hypotheses under $\boldsymbol{\theta} = (0.5, 0.5)$. ORD uses POC (top left), OBF (top right), TRIAN (bottom) boundary shapes for T_1 which control the type I error under $\boldsymbol{\theta} = (\infty, 0)$. Results are provided using 10^6 replications.

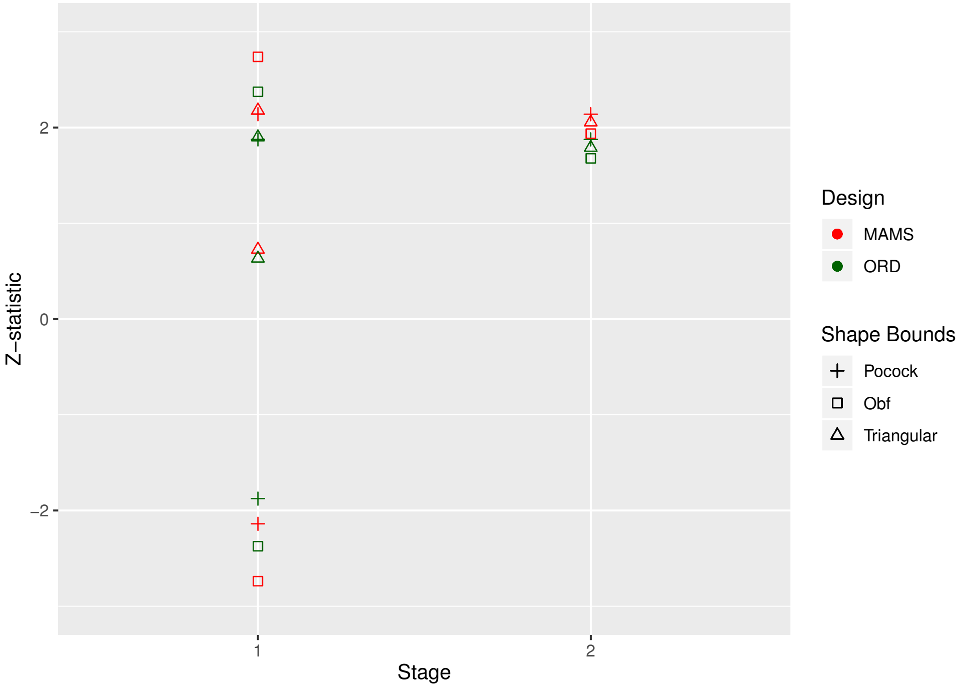

FIGURE 4 Values of the critical bounds under the global null hypothesis H_0 (when $\alpha = 0.05$) for 3-arm 2-stage ORD and standard MAMS[6] designs using Pocock bounds[2], O'Brien & Fleming[3] (Obf) and triangular[1] bounds for each treatment arm, $(u_j, l_j), j \in \{1, 2\}$.
